# Supplementary material for: Spatial entropy drives the maintenance and dissemination of transferable plasmids
Source: Mol Syst Biol. 2025 Apr 29;21(7):856–69. doi: 10.1038/s44320-025-00110-8 (PMC12222859; doi:10.1038/s44320-025-00110-8)
Supplement: Supplementary file 1 — Appendix [file 44320_2025_110_MOESM1_ESM.pdf]

APPENDIX

Spatial entropy drives the maintenance and dissemination of transferable plasmids

Wenzhi Xue[1*], Juken Hong[1*], Runmeng Zhao[2], Huaxiong Yao[1], Yi Zhang[1], Zhuojun Dai[1], Teng Wang[1]✉

[1]Key Laboratory of Quantitative Synthetic Biology, Shenzhen Institute of Synthetic Biology, Shenzhen Institute of Advanced Technology, Chinese Academy of Sciences, Shenzhen 518055, China

[2]School of Mathematics, Jilin University, Changchun 130012, China

* These authors contributed equally to the work

✉Correspondence: t.wang1@siat.ac.cn

Table of Contents

Appendix Text S1. Plasmid transfer dynamics in a simplified metacommunity composed of two patches 4
Appendix Text S2. Simualtion of plasmid dynamics in an array of patches undergoing periodic mixing 6
Appendix Text S3. Test of different function forms of the relationships between bacterial density and contact frequency 7
Appendix Text S4. Testing different spatial heterogeneity setups in patch simulations 8
Appendix Figure S1. The plasmid abundance accumulation rate as a function of its persistence potential. 10
Appendix Figure S2. Spatial entropy can be changed independently from the mean density of microbes in the metacommunity. 11
Appendix Figure S3. The total number of patches was not critical for the theoretical prediction. 12
Appendix Figure S4. The between-patch plasmid transfer rate ω was not critical for the prediction. 13
Appendix Figure S5. In a highly simplified metacommunity composed of only two patches, reducing spatial entropy continued to promote the abundance of the transferable plasmid. 14
Appendix Figure S6. In an array of patches connected by periodic mixing, reducing spatial entropy continues to promote plasmid maintenance. 15
Appendix Figure S7. The main prediction is robust to changes in the heterogeneity setup of the metacommunities. 16
Appendix Figure S8. The reaction order σ of plasmid transfer kinetics dictates the interplay between spatial entropy and plasmid maintenance. 17
Appendix Fig. S9 In metacommunities of *E. coli* strains undergoing periodic mixing, reducing spatial entropy promotes the effective transfer rate of the plasmid. 19
Appendix Figure S10. The distribution of plasmid numbers per genome in prokaryotes with or without biofilm related genes. 20
Appendix Figure S11. Carriage of BFGs on chromosomes is associated with enrichment of plasmids in prokaryotic genomes. 21
Appendix Figure S12. The positive correlation between BFG presence and plasmid carriage is independent of genome size effects. 22
Appendix Figure S13. The number of different types of resistance genes in the pool of prokaryotic genes. 23
Appendix Figure S14. Carriage of BFGs on chromosomes is associated with enrichment of ARGs in prokaryotic genomes. 24
Appendix Figure S15. BFG carriage enriches the IS elements in prokaryotic genomes. 25
Appendix Figure S16. BFG carriage enriches toxin/antitoxin related genes in prokaryotic genomes. 26
Appendix Figure S17. Biofilm formation remains positively correlated with the abundances of plasmids and ARGs in prokaryotic genomes, even with an expanded list of BFGs. 27
Appendix Figure S18. The majority of BFGs are carried by chromosomes instead of plasmids. 29
Appendix Figure S19. Biofilm formation remains positively correlated with the abundances of plasmids and ARGs in prokaryotic genomes, even when plasmids do not contribute to biofilm formation. 30
Appendix Table S1. The ratio between cell mixture and LB media in different wells 32
Appendix Table S2. List of biofilm formation-related genes curated from sequenced prokaryotic genomes 33
Appendix Table S3. List of biofilm formation-related genes curated from NCBI Gene database 37
Appendix Table S4. List of adhesion-related genes curated from NCBI Gene database 41

Appendix Table S5. List of exopolysaccharide production-related genes curated from NCBI Gene database.................42
Appendix Table S6. List of extracellular matrix-related genes curated from NCBI Gene database....................................43
Appendix Table S7. List of quorum sensing-related genes curated from NCBI Gene database...44

Appendix Text S1. Plasmid transfer dynamics in a simplified metacommunity composed of two patches

Consider the transfer of a plasmid in a metacommunity of two patches occupied by a single species. The dynamics of the system can be described by the following four ODEs:

$$\frac{ds_0}{dt} = \mu_0 s_0 \left(1 - \frac{s_0 + s_1}{N_m^s}\right) - \eta s_0 s_1 - \omega s_0 r_1 + \kappa s_1 - D s_0, \quad (1)$$

$$\frac{ds_1}{dt} = \mu_1 s_1 \left(1 - \frac{s_0 + s_1}{N_m^s}\right) + \eta s_0 s_1 + \omega s_0 r_1 - \kappa s_1 - D s_1, \quad (2)$$

$$\frac{dr_0}{dt} = \mu_0 r_0 \left(1 - \frac{r_0 + r_1}{N_m^r}\right) - \eta r_0 r_1 - \omega r_0 s_1 + \kappa r_1 - D r_0, \quad (3)$$

$$\frac{dr_1}{dt} = \mu_1 r_1 \left(1 - \frac{r_0 + r_1}{N_m^r}\right) + \eta r_0 r_1 + \omega r_0 s_1 - \kappa r_1 - D r_1. \quad (4)$$

Here, s_0 and s_1 are the densities of plasmid-free and plasmid-carrying cells in the first patch, respectively, while r_0 and r_1 are the densities of plasmid-free and plasmid-carrying cells in the second patch. μ_1 and μ_0 are their respective growth rates, with μ_1 related to μ_0 via $\mu_1 = \frac{\mu_0}{1+\lambda}$, where λ denotes the fitness effect of the plasmid. N_m^s and N_m^r are the maximum carrying capacities of the two patches. η is the within-patch plasmid transfer rate from donors to recipients. κ is the plasmid loss rate. D is the dilution rate. ω stands for the cross-patch transfer rate of the plasmid.

Let x be the proportion of N_m^s in the total carrying capacity: $x = \frac{N_m^s}{N_m^s + N_m^r}$. Following our general definition of spatial entropy, the entropy value of this simplified system can be calculated as:

$$\mathcal{H} = \frac{\exp\left[-x log x - (1-x)\log(1-x)\right]}{2}. \quad (5)$$

By changing x, we can examine the influence of entropy on plasmid abundance in this system. The results of numerical simulations are shown in Appendix Fig. S5.

Previous studies established a metric, termed plasmid persistence potential, that predicts the steady-state abundance of a transferable plasmid in the microbial population. Let s_1^* and r_1^* represent the steady-state abundances of plasmid-carrying cells in the two patches, respectively. The persistence potentials of the plasmid in the two patches (P_s and P_r, respectively) can be defined as:

$$P_s = \frac{\eta + \omega \frac{r_1^*}{s_1^*}}{\Delta}, \quad (6)$$

$$P_r = \frac{\eta + \omega \frac{s_1^*}{r_1^*}}{\Delta}. \quad (7)$$

Here, Δ is a combination of dilution rate, plasmid burden and segregation loss rate:

$$\Delta = \frac{\mu_0}{\mu_0 - D}\left(D + \kappa - \frac{D}{1+\lambda}\right), \quad (8)$$

where λ describes the fitness burden of the plasmid.

Persistence potential determines the steady-state abundace of a plasmid in the local population. The relationship between these two quantities can be approximated by:

$$\frac{s_1^*}{N_m^s} = 1 - \frac{1}{P_s}, \quad (9)$$

$$\frac{r_1^*}{N_m^r} = 1 - \frac{1}{P_r}. \quad (10)$$

Given definitions of P_s and P_r in equations (6) and (7), the steady-state abundances of the plasmid in the two patches can be obtained by solving the following two equations:

$$\frac{s_1^*}{N_m^s} = 1 - \frac{\Delta}{\eta + \omega \frac{r_1^*}{s_1^*}}, \quad (11)$$

$$\frac{r_1^*}{N_m^r} = 1 - \frac{\Delta}{\eta + \omega \frac{s_1^*}{r_1^*}}. \quad (12)$$

These two equations can be analytically solved, although the formulation of the solution is highly complicated:

$$\begin{cases} s_1^* = \dfrac{[(\frac{\eta}{\Delta})^2 - (\frac{\omega}{\Delta})^2]\hbar^2}{\frac{\omega}{\Delta}N_m^r} - \dfrac{N_m^s(\frac{\eta}{\Delta})^2 - 2N_m^s\frac{\eta}{\Delta} - N_m^s(\frac{\omega}{\Delta})^2 + N_m^s}{\frac{\eta}{\Delta}} - \dfrac{\hbar[N_m^r(\frac{\eta}{\Delta})^3 - N_m^s(\frac{\eta}{\Delta})^2\frac{\omega}{\Delta} - N_m^r(\frac{\eta}{\Delta})^2 - N_m^r\frac{\eta}{\Delta}(\frac{\omega}{\Delta})^2 + N_m^s\frac{\eta}{\Delta}\frac{\omega}{\Delta} + N_m^s(\frac{\omega}{\Delta})^3 + N_m^r(\frac{\omega}{\Delta})^2]}{\frac{\eta}{\Delta}\frac{\omega}{\Delta}N_m^r}, & (13) \\ r_1^* = \hbar. & (14) \end{cases}$$

Here h is the solution of the cubic equation:

$$\begin{aligned} &\tfrac{\eta}{\Delta}(\tfrac{\omega}{\Delta})^2\hbar^3 - (\tfrac{\eta}{\Delta})^3\hbar^3 - 2\tfrac{\eta}{\Delta}(\tfrac{\omega}{\Delta})^2 N_m^r\hbar^2 + \tfrac{\eta}{\Delta}\tfrac{\omega}{\Delta}N_m^s\hbar^2 - (\tfrac{\eta}{\Delta})^2\tfrac{\omega}{\Delta}N_m^s\hbar^2 + (\tfrac{\omega}{\Delta})^3 N_m^s\hbar^2 + (\tfrac{\omega}{\Delta})^2 N_m^r\hbar^2 + 2(\tfrac{\eta}{\Delta})^3 N_m^r\hbar^2 \\ &\quad - 2(\tfrac{\eta}{\Delta})^2 N_m^r\hbar^2 - 3\tfrac{\eta}{\Delta}\tfrac{\omega}{\Delta}N_m^s N_m^r\hbar + 2(\tfrac{\eta}{\Delta})^2\tfrac{\omega}{\Delta}N_m^s N_m^r\hbar + \tfrac{\eta}{\Delta}(\tfrac{\omega}{\Delta})^2(N_m^r)^2\hbar - 2(\tfrac{\omega}{\Delta})^3 N_m^s N_m^r\hbar \\ &\quad + \tfrac{\omega}{\Delta}N_m^s N_m^r\hbar + 2(\tfrac{\eta}{\Delta})^2(N_m^r)^2\hbar - (\tfrac{\omega}{\Delta})^2(N_m^r)^2\hbar - (\tfrac{\eta}{\Delta})^3(N_m^r)^2\hbar - \tfrac{\eta}{\Delta}(N_m^r)^2\hbar + 2\tfrac{\eta}{\Delta}\tfrac{\omega}{\Delta}N_m^s(N_m^r)^2 \\ &\quad - (\tfrac{\eta}{\Delta})^2\tfrac{\omega}{\Delta}N_m^s(N_m^r)^2 + (\tfrac{\omega}{\Delta})^3 N_m^s(N_m^r)^2 - \tfrac{\omega}{\Delta}N_m^s(N_m^r)^2 = 0. \quad (15) \end{aligned}$$

The relative abundance of the plasmid in the metacommunity can then be approximately calculated by $\frac{s_1^*+r_1^*}{N_m^s+N_m^r}$. Let x be the proportion of N_m^s in the total carrying capacity: $x = \frac{N_m^s}{N_m^s+N_m^r}$. The spatial entropy

of this simplified system can be calculated as $\mathcal{H} = \frac{\exp[-x log x - (1-x)\log(1-x)]}{2}$. In this way, the relationship between spatial entropy and plasmid abundance can be analytically derived.

Appendix Text S2. Simualtion of plasmid dynamics in an array of patches undergoing periodic mixing

To investigate whether spatial entropy continues to affect plasmid persistence in metacommunities undergoing periodic mixing, we considered an array of patches. The system dynamics were modeled as a series of repeated cycles, each consisting of two alternating stages: mixing and growth.

During the mixing stage, a fraction f of the local population in each patch was replaced by populations from adjacent patches. After mixing, local populations in each patch grew independently without further cross-patch dispersal until the next mixing event. To align with our experimental setup, where cell densities were directly set, we assumed constant population density within each patch during the growth stage. The population dynamics during this stage can be described by the following equations:

$$\frac{ds_0^{(n)}}{dt} = -\eta s_0^{(n)} s_1^{(1)} + \varpi s_1^{(n)}, \quad (16)$$

$$\frac{ds_1^{(n)}}{dt} = \eta s_0^{(n)} s_1^{(1)} - \varpi s_1^{(n)}. \quad (17)$$

Here, $s_0^{(n)}$ and $s_1^{(n)}$ represent the densities of plasmid-free and plasmid-carrying cells in the n-th patch, respectively. η is the plasmid transfer rate. ϖ is the combined plasmid loss rate. Within each patch, the total density of $s_0^{(n)}$ and $s_1^{(n)}$ remains constant:

$$\frac{ds_0^{(n)}}{dt} + \frac{ds_1^{(n)}}{dt} = 0. \quad (18)$$

At the end of each cycle, each patch partially mixes with its neighboring patches, creating a new initial condition for the next cycle.

To control the spatial entropy of the system, we randomized the density distributions among different patches and then simulated the plasmid dynamics over subsequent cycles. After a certain number of mixing events, we calculated both the spatial entropy of the metacommunity and the overall plasmid abundance across all patches. Our results consistently showed that reducing spatial entropy enhanced plasmid abundance in this system, thereby confirming the robustness of our prediction.

Appendix Text S3. Test of different function forms of the relationships between bacterial density and contact frequency

We assumed a quadratic relationship between the effective transfer efficiency and local density. This relationship can be derived from the basic kinetics of plasmid transfer. Let D, R and T represent the densities of plasmid donors, recipients and transconjugants, respectively. The reaction order (denoted as σ) of plasmid transfer describes the relationship between the rate of transconjugant production and the concentrations of donors and recipients:

$$\frac{d[T]}{dt} = \eta[D]^{\sigma}[R]^{\sigma}. \quad (19)$$

Let C be the overall cell density in the local population, and ϑ be the ratio between $[D]$ and $[R]$. The rate of plasmid transfer can then be expressed as:

$$\frac{d[T]}{dt} = \eta(\frac{\vartheta}{1+\vartheta})^{\sigma}(\frac{1}{1+\vartheta})^{\sigma}[C]^{2\sigma}. \quad (20)$$

Therefore, the relationship between transfer efficiency and cell density is determined by the value of σ:

$$\frac{d[T]}{dt} \propto [C]^{2\sigma}. \quad (21)$$

The value of σ dictates whether the gain of transfer efficiency from increased density outweighs the loss from local density reduction. Specifically, when $\sigma > 0.5$, density increases in certain regions can compensate for decreased transfer rates elsewhere. When $\sigma = 0$, corresponding to zero-order kinetics, the relationship between transfer efficiency and cell density saturates.

Classic plasmid ecological theories commonly employ a mass-action model, where single cells randomly collide and exchange plasmids upon collision, leading to first-order kinetics of plasmid transfer ($\sigma = 1$). In our simulations, we also assumed $\sigma = 1$, consistent with previous studies. Therefore, the plasmid transfer efficiency becomes quadratically related to cell density:

$$\frac{d[T]}{dt} \propto [C]^{2}. \quad (22)$$

In this case, the gain in transfer efficiency from increased density exceeds the loss from local density reduction.

We have conducted additional simulations to evaluate how our general conclusions are influenced by varying the relationship between transfer efficiency and cell density. Specifically, we repeated the patch dynamics simulations under three conditions for σ:

(1) When $\sigma < 0.5$: The gain in contact frequency from increased density does not compensate for the loss from local density reduction. In this case, reducing spatial entropy slows down the accumulation rate of a transferable plasmid (Appendix Fig. S8A).

(2) When $\sigma = 0.5$: The gain in contact frequency from increased density equals the loss from local

density reduction. In this case, reducing spatial entropy does not influence the accumulation rate of a transferable plasmid (Appendix Fig. S8B).

(3) When $\sigma > 0.5$: The gain in contact frequency from increased density exceeds the loss from local density reduction. In this case, reducing spatial entropy promotes the accumulation rate of a transferable plasmid (Appendix Fig. S8C and D).

We also considered a scenario where cell contact frequency is a Hill function of bacterial density (Appendix Fig. S8E). In this case, the reaction order σ of plasmid transfer transitions from 1 to 0 as density increases. Our simulation results suggested that reducing spatial entropy slows down the accumulation rate of a transferable plasmid in this scenario.

These results highlight that the functional form of the density-bacterial contact frequency relationship indeed affects the interplay between spatial entropy and plasmid maintenance. While various mathematical forms can be explored, the general conclusion that 'spatial heterogeneity promotes plasmid maintenance', validated by experiments and bioinformatic analysis, suggested that $\sigma > 0.5$ is more realistic. This assumption is also consistent with classic plasmid ecological theories which commonly use $\sigma = 1$ for plasmid transfer kinetics.

Appendix Text S4. Testing different spatial heterogeneity setups in patch simulations

In this study, we employed Perlin noise-like distributions to model the diverse spatial landscapes of metacommunities. To determine whether the relationship between spatial entropy and plasmid maintenance is dependent on specific heterogeneity setups, we tested additional spatial structures generated using 2D Gaussian and 2D uniform distribution algorithms.

In a metacommunity of $m \times m$ patches, we generated spatial structures from Gaussian distributions by first randomizing the number of peaks (n_{peak}) in the landscape. The location of each peak was also randomized as $[u_{x,i}, u_{y,i}]$ (where $1 \le i \le n_{peak}$). Here, $u_{x,i}$ and $u_{y,i}$ are integers between 1 and m. For the i-th peak, we generated a 2D Gaussian function:

$$f_i(x,y) = \frac{1}{2\pi\sigma_i^2} e^{-\frac{(x-u_{x,i})^2+(y-u_{y,i})^2}{2\sigma_i^2}}. \quad (23)$$

Here, σ_i is the standard deviation corresponding to the i-th peak. In the simulations, we randomized σ_i between 3 and $\frac{m}{2} + 3$.

With all $f_i(x,y)$ defined (for $1 \le i \le n_{peak}$), we generated a landscape represented by a $m \times m$ matrix $[a_{x,y}]$ (where $1 \le x, y \le m$):

$$a_{x,y} = \sum_{i=1}^{n_{peak}} f_i(x,y). \quad (24)$$

We calculated the maximum carrying capacity N_m at the patch $[i, j]$ as $z\frac{|a_{ij}|^n}{\sum_{i,j}|a_{ij}|^n}\cdot m^2$, such that the mean value of N_m across all the patches equaled z. The mean cell density in the metacommunity can be controlled by adjusting z. Without loss of generality, we set $z = 1$ in our numerical simulations. By randomizing n_{peak}, $u_{x,i}$, $u_{y,i}$ and σ_i, we generated diverse landscapes with various spatial entropies (Appendix Fig. S7A and C).

Similarly, to generate spatial structures from uniform distributions, we first randomized the number of centers (n_{center}) in the landscape. The location of each center was also randomized as $[u_{x,i}, u_{y,i}]$ (where $1 \leq i \leq n_{center}$). $u_{x,i}$ and $u_{y,i}$ are integers between 1 and m. Each center is surrounded by an interval and corresponds to a uniform distribution function:

$$g_i(x, y) = \begin{cases} 0, & \text{for } x - u_{x,i} > \sigma_i \text{ or } y - u_{y,i} > \sigma_i, \\ h_i, & \text{for } x - u_{x,i} \leq \sigma_i \text{ and } y - u_{y,i} \leq \sigma_i. \end{cases} \quad (25)$$

σ_i characterizes the width of each interval. In the simulations, we randomized σ_i between 1 and $\frac{m}{2} + 1$ following a uniform distribution.

With all $g_i(x, y)$ defined (for $1 \leq i \leq n_{center}$), we generated a landscape represented by a $m \times m$ matrix $[a_{x,y}]$ (where $1 \leq x, y \leq m$):

$$a_{x,y} = \sum_{i=1}^{n_{center}} g_i(x, y). \quad (26)$$

We calculated the maximum carrying capacity N_m at the patch $[i, j]$ as $z\frac{|a_{i,j}|^n}{\sum_{i,j}|a_{i,j}|^n}\cdot m^2$, such that the mean value of N_m's in all the patches equaled z. By randomizing n_{center}, $u_{x,i}$, $u_{y,i}$, h_i and σ_i, we generated diverse landscapes with various spatial entropies (Appendix Fig. S7B and D).

Appendix Figures

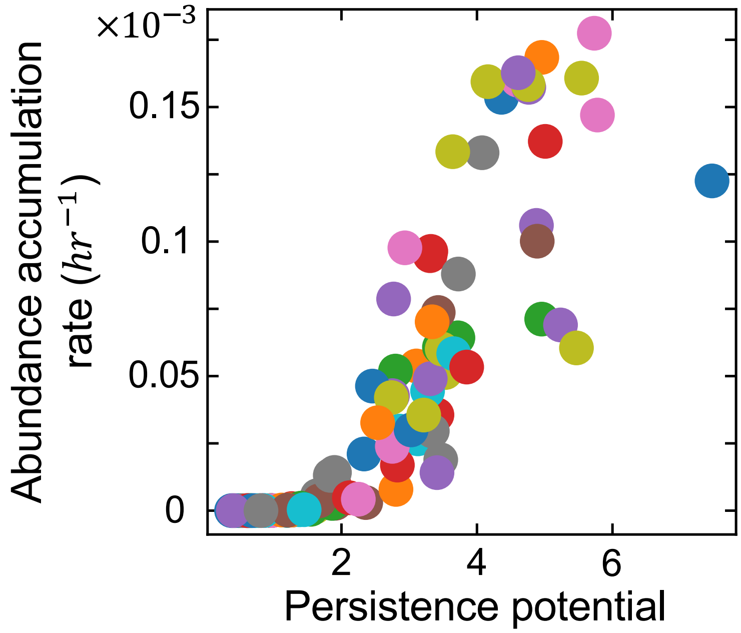

Appendix Figure S1. The plasmid abundance accumulation rate as a function of its persistence potential.

The simulation was performed on a landscape created by Perlin noise generator. We randomized the parameters 100 times following uniform distributions in the ranges of $0.01 < \eta < 0.1\ hr^{-1}$, $0.01 < \kappa < 0.02\ hr^{-1}$, $0.3 < \mu_1 < 0.5\ hr^{-1}$, $0.01 < D < 0.05\ hr^{-1}$. Other parameters were $\mu_0 = 0.5\ hr^{-1}$ and $\omega = 0.04\ hr^{-1}$. Persistence potential was calculated as $\eta/(D + \kappa - \frac{D}{1+\lambda})$.

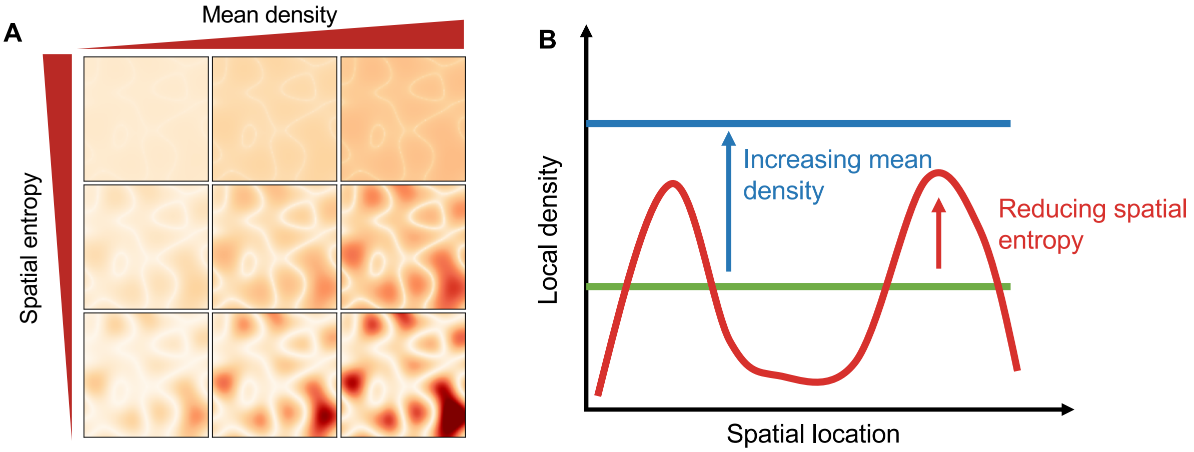

Appendix Figure S2. Spatial entropy can be changed independently from the mean density of microbes in the metacommunity.

(A) Examples of landscapes with different mean densities and spatial entropies. Three values of mean densities (0.5, 1 and 1.5 from left to right) were tested. The data utilized to generate the middle column were identical to those employed in the middle column of Figure 3A.

(B) A schematic of how entropy and mean density can be controlled independently from each other. Entropy is associated with the extent of microbial density variations across space, instead of the average density. Reducing entropy creates not only areas with higher local densities, but also areas with lower densities.

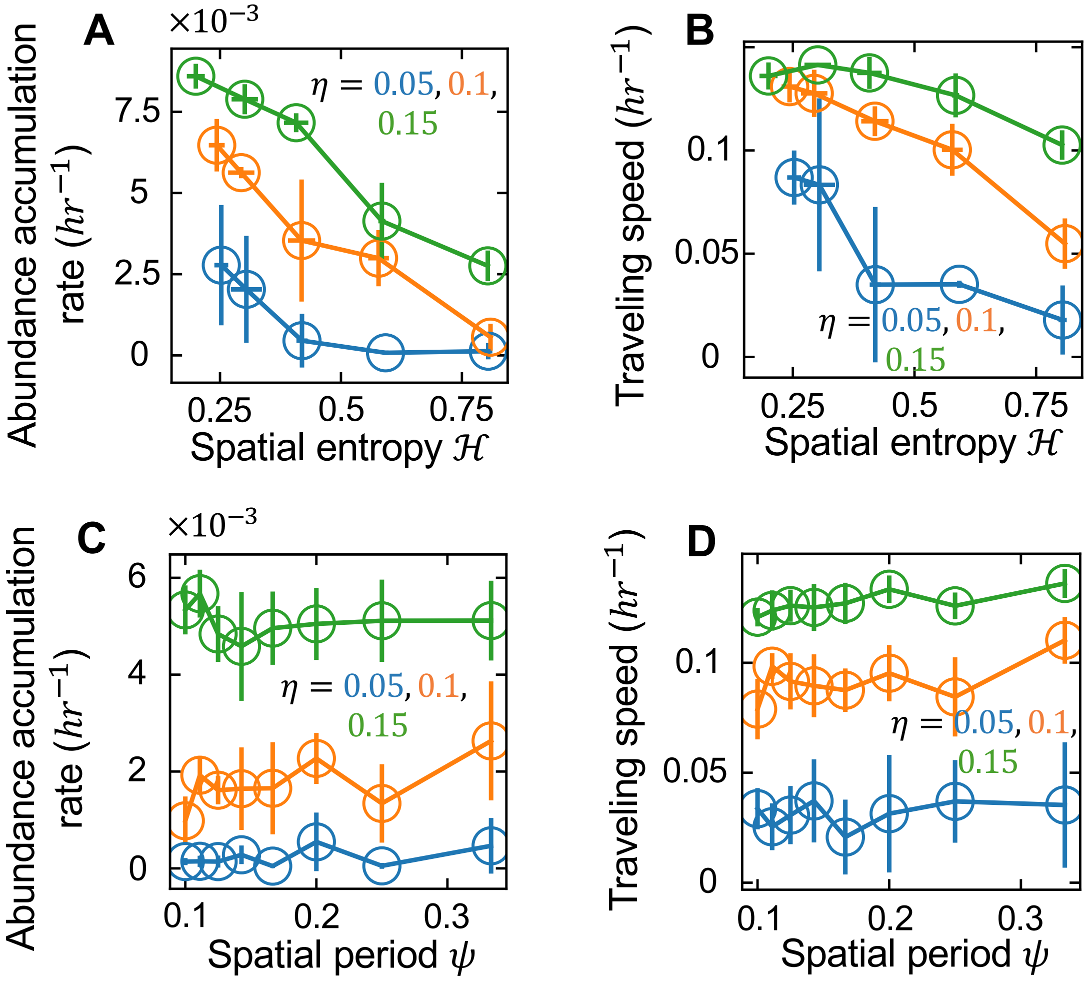

Appendix Figure S3. The total number of patches was not critical for the theoretical prediction.

(A-B) Reducing spatial entropy sped up the accumulation and dissemination of a transferable plasmid. Here, numerical simulations were performed in metacommunities of 21×21 patches. Three different η values were tested and shown as examples. Other parameters were $\mu_0 = 0.5\ hr^{-1}$, $\mu_1 = 0.48\ hr^{-1}$, $\kappa = 0.01\ hr^{-1}$, $D = 0.02\ hr^{-1}$, $\omega = 0.04\eta$. Data were presented as mean $\pm$ standard deviation of 5 replicates. Each replicate represented a unique spatial landscape generated by Perlin noise.

(C-D) Spatial periodicity didn't significantly change the accumulation and dissemination of the plasmid. Numerical simulations were performed in metacommunities of 21×21 patches. Periodicity was controlled by the octave parameter in the Perlin noise algorithm. Eight different octave values, from 3 to 10, were tested. Data were presented as mean $\pm$ standard deviation of 5 replicates.

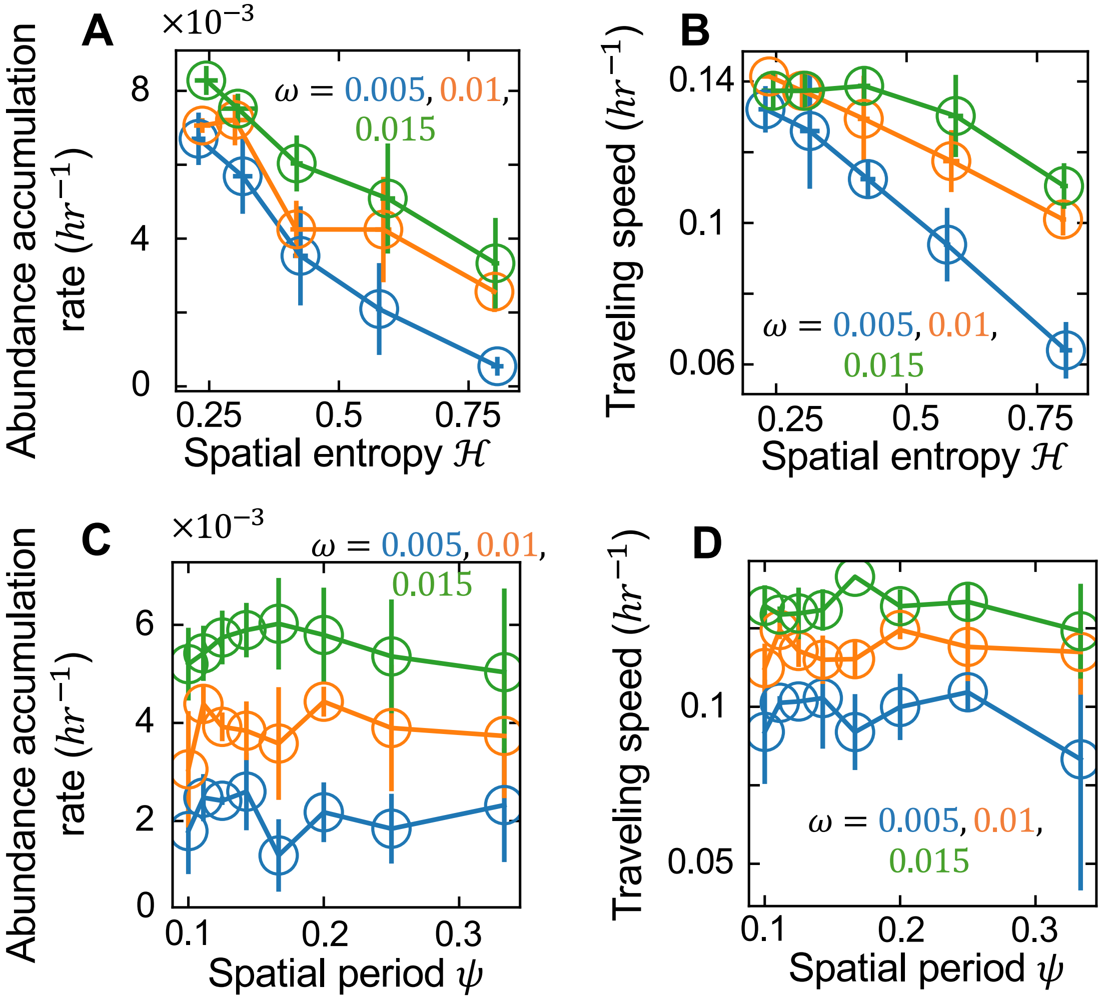

Appendix Figure S4. The between-patch plasmid transfer rate ω was not critical for the prediction.

(A-B) Reducing spatial entropy sped up the accumulation and dissemination of the plasmid. Here, numerical simulations were performed in metacommunities of 21×21 patches. Three different ω values were tested and shown as examples. Other parameters were $\mu_0 = 0.5\ hr^{-1}$, $\mu_1 = 0.48\ hr^{-1}$, $\kappa = 0.01\ hr^{-1}$, $D = 0.02\ hr^{-1}$, $\eta = 0.1\ hr^{-1}$. Data were presented as mean $\pm$ standard deviation of 5 replicates.

(C-D) Spatial periodicity didn't significantly change the accumulation and dissemination of the plasmid. Periodicity was controlled by the octave parameter in the Perlin noise algorithm. Eight different octave values, from 3 to 10, were tested. Data were presented as mean $\pm$ standard deviation of 5 replicates.

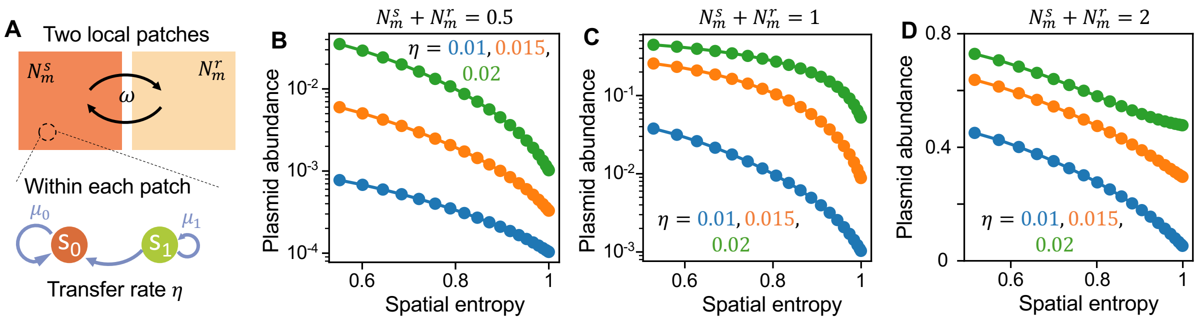

Appendix Figure S5. In a highly simplified metacommunity composed of only two patches, reducing spatial entropy continued to promote the abundance of the transferable plasmid.

(A) Schematic of the simplified system. In each patch, s_0 and s_1 represent the abundances of plasmid-free and plasmid-carrying cells, respectively. μ_0 and μ_1 are their respective growth rates. N_m^s and N_m^r denote the maximum carrying capacities of the two patches. η and ω describe the within-patch and across-patch plasmid transfer rates, respectively.

(B-D) Reducing spatial entropy promotes plasmid abundance. Three different values of $N_m^s + N_m^r$ were tested (from left to right). Spatial entropy was adjusted by controlling the ratio between N_m^s and N_m^r. For each value of $N_m^s + N_m^r$, three different η values were tested and shown as examples. Other parameters were $\mu_0 = 0.5\ hr^{-1}$, $\mu_1 = 0.48\ hr^{-1}$, $\kappa = 0.01\ hr^{-1}$, $D = 0.02\ hr^{-1}$, $\omega = 0.04\eta$.

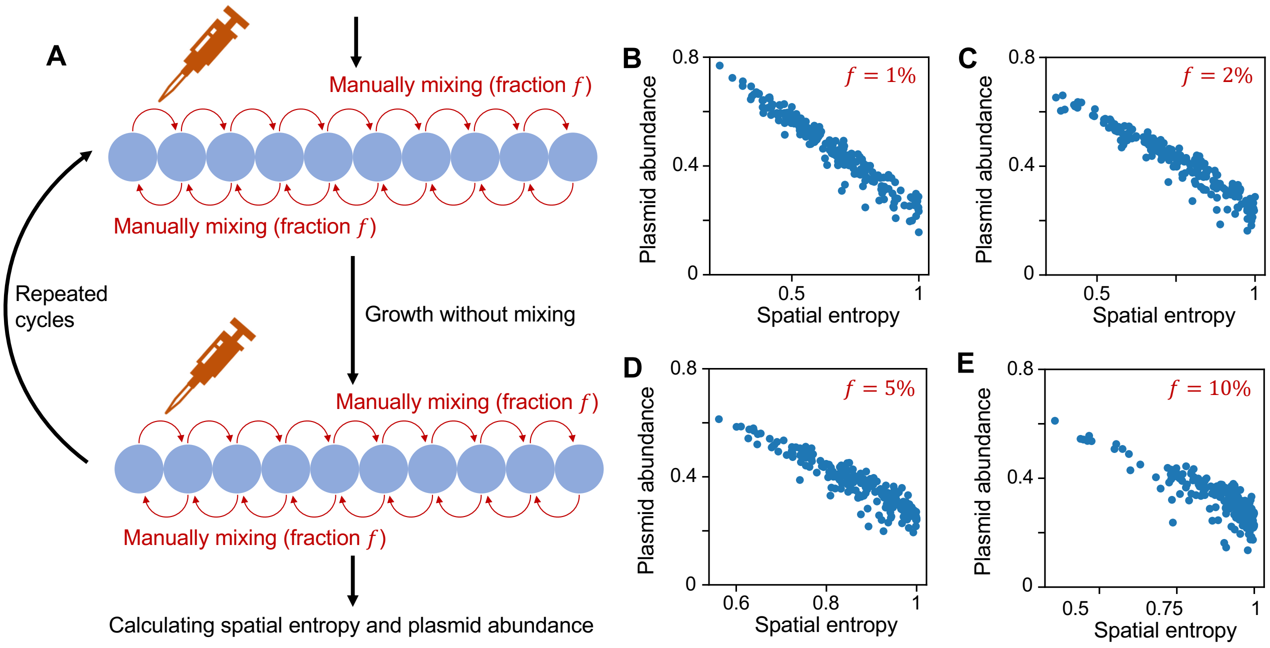

Appendix Figure S6. In an array of patches connected by periodic mixing, reducing spatial entropy continues to promote plasmid maintenance.

(A) Schematic of a metacommunity undergoing periodic mixing events. During mixing, a fraction (f) of the local population in each patch is replaced by populations from adjacent patches. After mixing, local populations in each patch grow independently without cross-patch dispersal until the next mixing event. We controlled the spatial entropy of the system by altering the density distributions. After a certain number of mixing events, we calculated both the spatial entropy of the metacommunity and the overall plasmid abundance across different patches.

(B-E) Reducing entropy promoted plasmid persistence and abundance. The initial densities of different patches were randomized to generate a variety of entropies. The metacommunity then underwent repeated cycles of mixing and growth. The spatial entropy and overall plasmid abundance were calculated after 20 cycles. Four different f values were tested and shown as examples (from left to right). Other parameters are $\eta = 0.02\ hr^{-1}$ and $\varpi = 0.01\ hr^{-1}$.

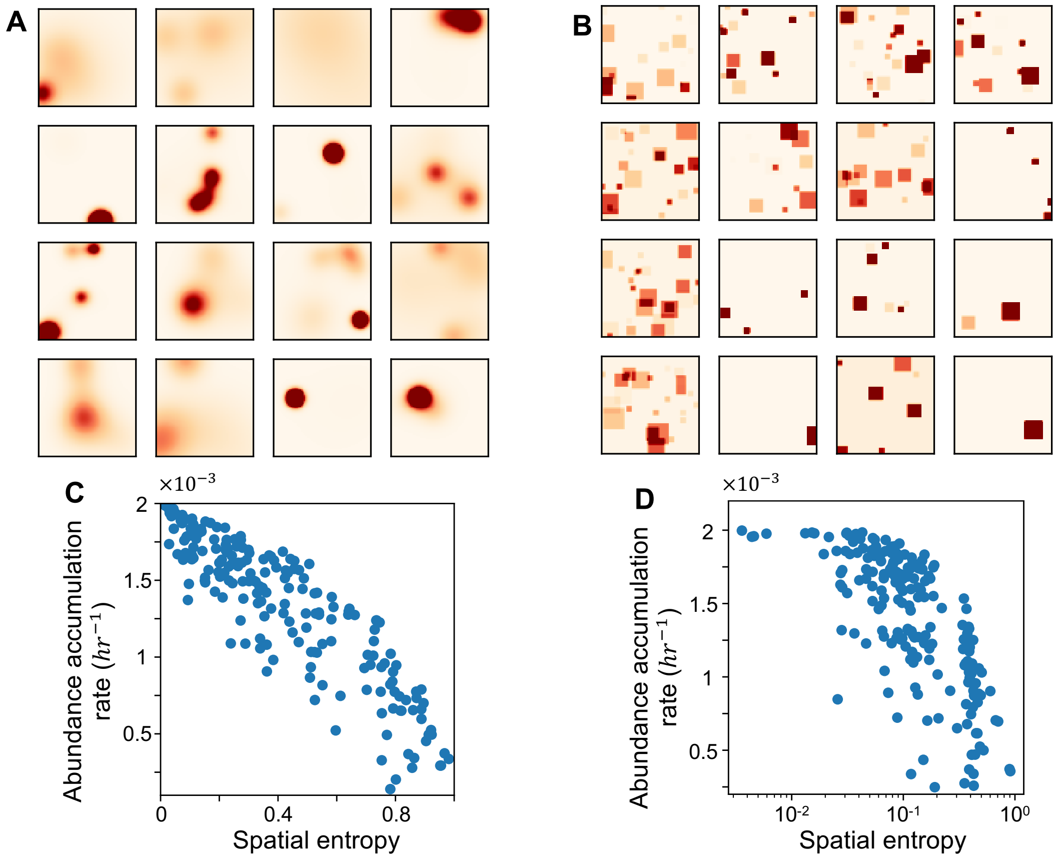

Appendix Figure S7. The main prediction is robust to changes in the heterogeneity setup of the metacommunities.

(A) Examples of spatial landscapes created by 2D Gaussian distribution generators. Each metacommunity containes 51×51 patches. The cell densities in different patches were determined by a random number of 2D Gaussian distributions. The peak position, the peak value and the standard deviation of each Gaussian distribution were all randomized, leading to large diversity of spatial landscapes.

(B) Plasmid accumulation rate is negatively associated with spatial entropy in landscapes created by 2D Gaussian distribution generators. 200 randomized landscapes were created, and for each landscape, a transferable plasmid was seeded in the central patch. Then the plasmid transfer dynamics was simulated. The accumaltion rates of plasmid abundance in the first 500 hours were plotted against the spatial entropy of the landscape. Other parameters were $\mu_0 = 0.5\ hr^{-1}$, $\mu_1 = 0.45\ hr^{-1}$, $\kappa = 0.01\ hr^{-1}$, $D = 0.02\ hr^{-1}$, $\eta = 0.05\ hr^{-1}$, $\omega = 0.04\eta$.

(C) Examples of spatial landscapes created by 2D uniform distribution generators. Each metacommunity containes 51×51 patches. The cell densities in different patches were determined by a random number of 2D uniform distributions.

(D) Plasmid accumulation rate is negatively associated with spatial entropy in landscapes created by 2D uniform distribution generators.

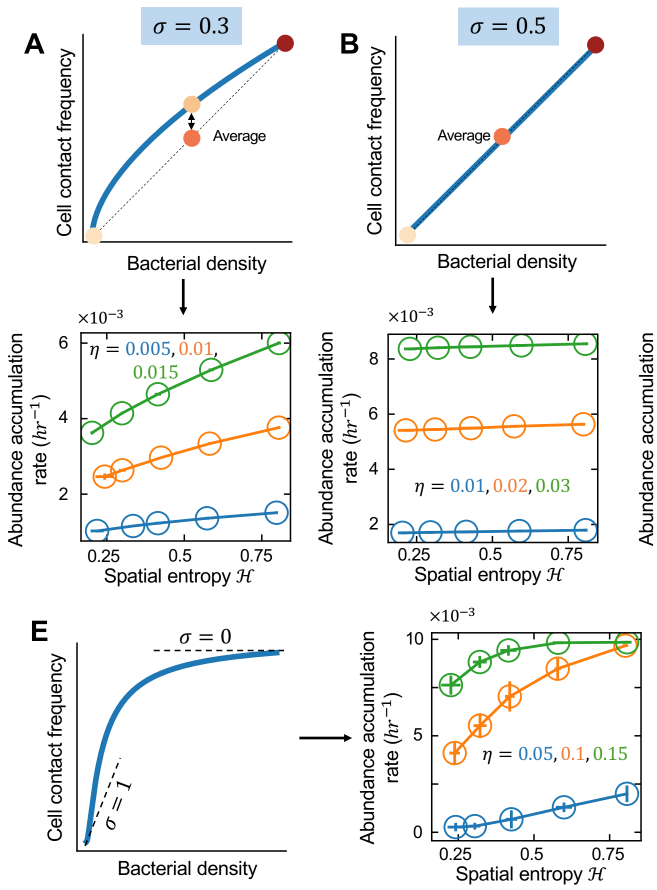

Appendix Figure S8. The reaction order σ of plasmid transfer kinetics dictates the interplay between spatial entropy and plasmid maintenance.

(A) When $\sigma = 0.3$, the gain in contact frequency from density increase fails to compensate for the loss from local density reduction (top panel). Therefore, the average cell contact frequency increases with entropy. Consequently, reducing spatial entropy slows down the accumulation rate of a transferable plasmid (bottom panel). Numerical simulations were performed in metacommunities of 21×21 patches. Three different η values were tested and shown as examples. Other parameters were $\mu_0 = 0.5\ hr^{-1}$, $\mu_1 = 0.48\ hr^{-1}$, $\kappa = 0.01\ hr^{-1}$, $D = 0.02\ hr^{-1}$, $\omega = 0.04\eta$. Data were presented as mean $\pm$ standard deviation of 5 replicates, each representing a unique spatial landscape generated by Perlin noise.

(B) When $\sigma = 0.5$, the gain in contact frequency by density increase equals the loss from local density reduction (top panel). Therefore, the average cell contact frequency is unaffected by entropy change. In this case, reducing spatial entropy does not influence the accumulation rate of a transferable plasmid (bottom panel). Data were presented as mean $\pm$ standard deviation of 5 replicates.

(C) When $\sigma = 0.8$, the gain in contact frequency by density increase exceeds the loss from local density reduction (top panel). Therefore, the average cell contact frequency is elevated by entropy reduction. In this case, reducing spatial entropy promotes the accumulation rate of a transferable plasmid (bottom panel). Data were presented as mean $\pm$ standard deviation of 5 replicates.

(D) When $\sigma = 1$, the gain in contact frequency by density increase exceeds the loss from local density reduction (top panel). Therefore, the average cell contact frequency is elevated by entropy reduction. Consequently, reducing spatial entropy promotes the accumulation rate of a transferable plasmid (bottom panel). Data were presented as mean $\pm$ standard deviation of 5 replicates.

(E) When cell contact frequency is a hill function of bacterial density, the reaction order σ of plasmid transfer transitions from 1 to 0 when density increases (left panel). When bacterial density is sufficiently high, cell contact frequency saturates. In this case, the gain in contact frequency from increased density fails to compensate for the loss from local density reduction. Thus, reducing entropy decreases the average cell contact frequency (right panel). Consequently, reducing spatial entropy slows down the accumulation rate of a transferable plasmid. Data are presented as mean $\pm$ standard deviation of 5 replicates.

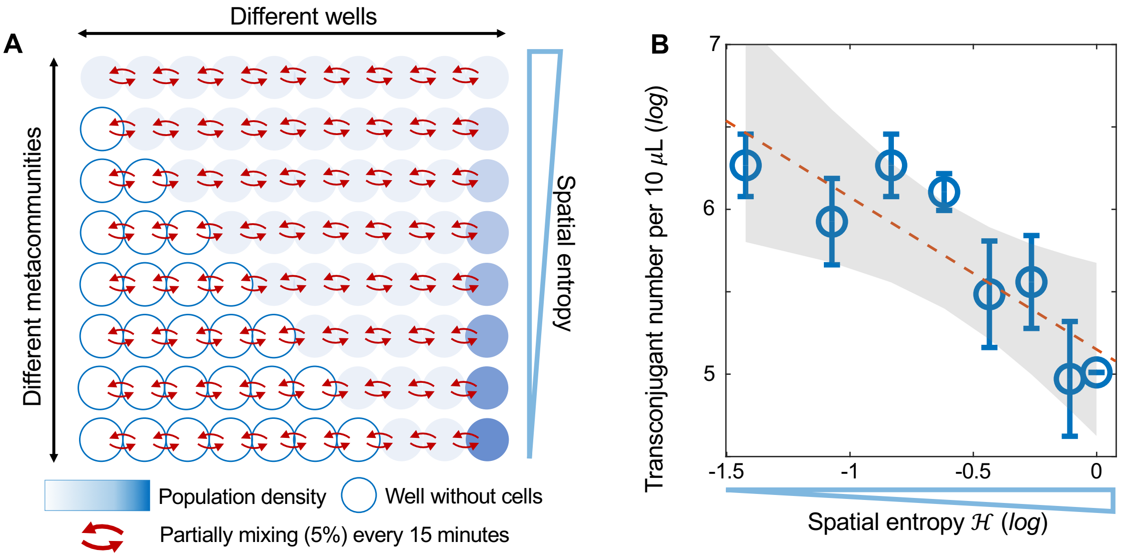

Appendix Fig. S9 In metacommunities of *E. coli* strains undergoing periodic mixing, reducing spatial entropy promotes the effective transfer rate of the plasmid.

(A) Schematic of the experimental design. Donor cells carrying the F plasmid were mixed with the recipients. The mixture was then allocated into different rows of wells. Each row contained the same total number of cells, but the population distribution among the wells varied, creating a range of spatial entropies. Every 15 minutes, 10 μL of culture was taken from every well and transferred to its neighboring wells, mimicking microbial dispersal across patches. After one hour of incubation, the total number of transconjugants within each group was quantified by selective plating.

(B) Reducing spatial entropy promotes the total number of transconjugants (per 10 μL) in each metacommunity. Since the total amounts of donors or recipients were equal across different groups, the number of transconjugants in each group reflects the effective transfer rate (η_{eff}). Data were presented as mean $\pm$ standard deviation of 3 replicates. The linear regression between log(transconjugant number) and log(spatial entropy) is shown as a dashed line, with the shaded area representing the 95% confidence interval.

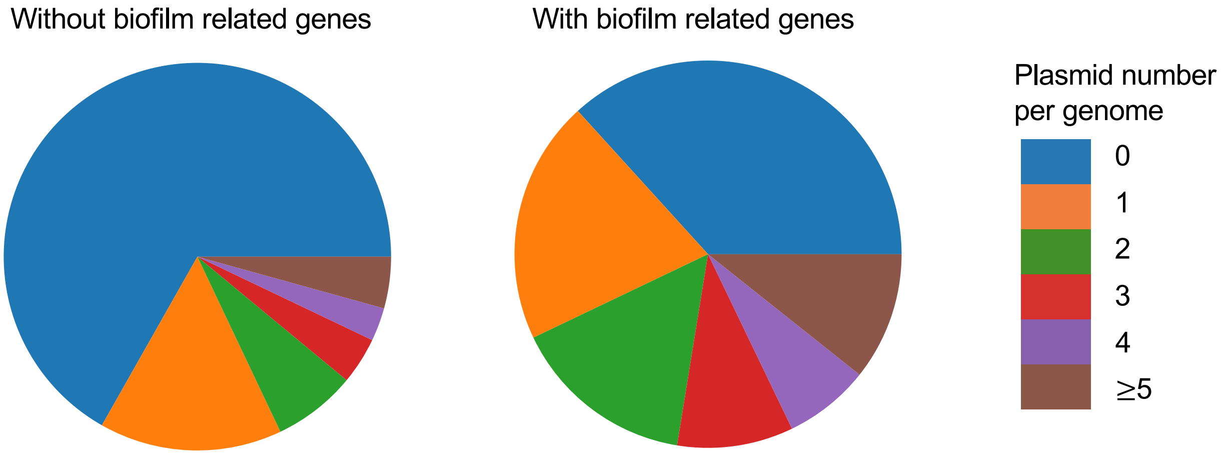

Appendix Figure S10. The distribution of plasmid numbers per genome in prokaryotes with or without biofilm related genes.

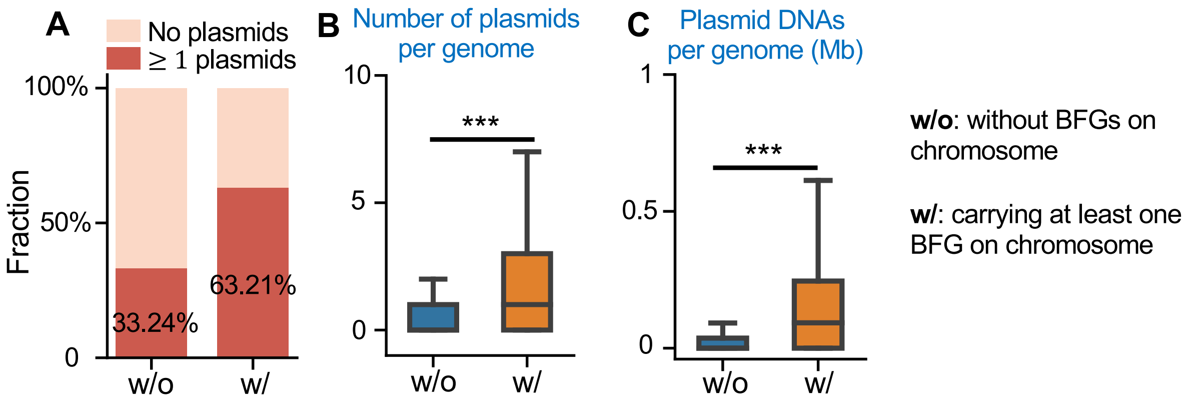

Appendix Figure S11. Carriage of BFGs on chromosomes is associated with enrichment of plasmids in prokaryotic genomes.

(A) 33.24% of the genomes without chromosomal BFGs carry plasmids. For genomes with chromosomal BFGs, this fraction increases to 63.21%.

(B) Genomes with chromosomal BFGs in general carry greater number of plasmids compared with those without chromosomal BFGs. The triple asterisk notation (***) denotes statistical significance at the $p < 0.001$ level, as determined by two-sided Student's t-tests.

(C) Genomes with chromosomal BFGs carry greater amounts of plasmid DNAs compared with those without chromosomal BFGs. Here, the amount of plasmid DNAs was calculated by summing the sizes of all different plasmids in the genome.

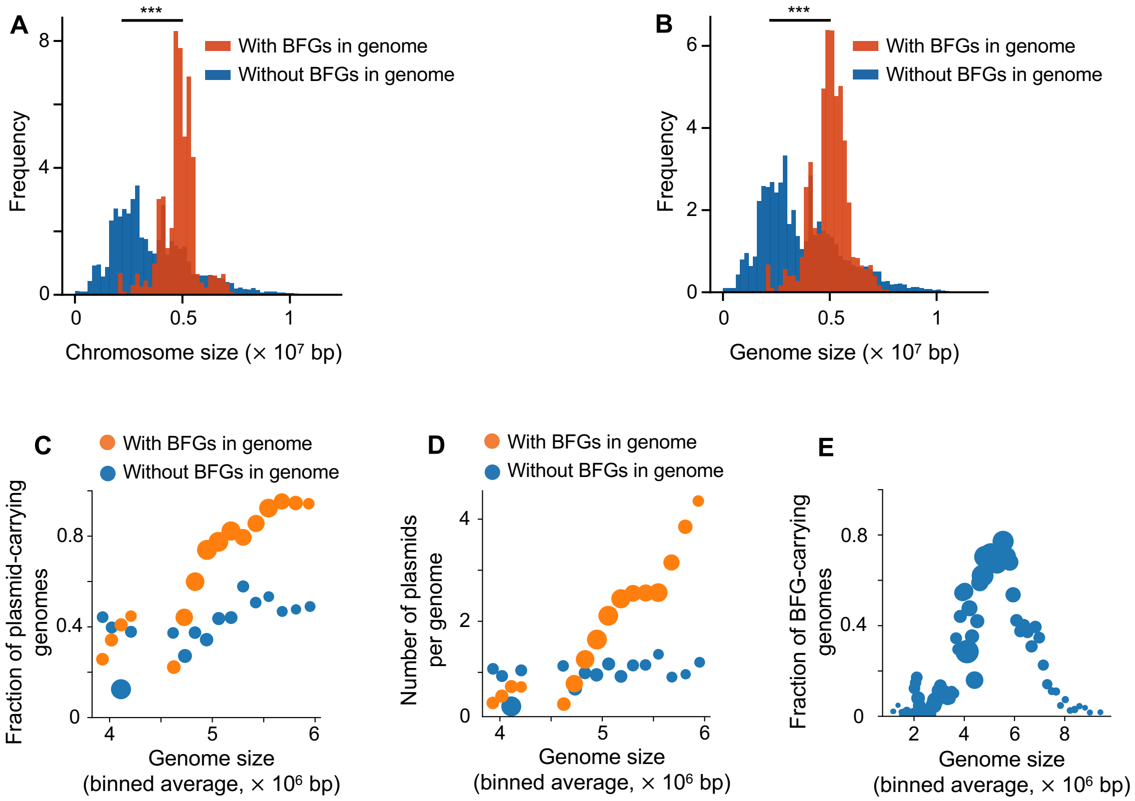

Appendix Figure S12. The positive correlation between BFG presence and plasmid carriage is independent of genome size effects.

(A) Prokaryotic chromosomes with BFGs were significantly larger than those without BFGs. The triple asterisk notation (***) denotes statistical significance at the $p < 0.001$ level, as determined by two-sided Student's t-tests.

(B) Prokaryotic genomes with BFGs were significantly larger than those without BFGs.

(C) BFG presence leads to a higher frequency of plasmid carriage even when genome size is controlled. We divided the collection of prokaryotic genomes into multiple bins based on genome size, ensuring that the size difference between the largest and smallest genomes within each bin was less than 2.4%. Within each bin, genomes were further divided into two groups: those with BFGs and those without. The fraction of plasmid-carrying genomes was calculated for each group. Data are shown only for groups containing at least 200 genomes; bins with fewer genomes were excluded due to insufficient data. Marker size represents the number of genomes in each group.

(D) BFG presence leads to a greater number of plasmids in prokaryotic genomes even when genome size is controlled.

(E) The relationship between the fraction of BFG-carrying genomes and genome size. We divided the collection of prokaryotic genomes into multiple bins based on genome size and calculated the fraction of BFG-carrying genomes within each bin. This fraction was plotted against the binned-average genome size. Marker size represents the number of genomes in each bin.

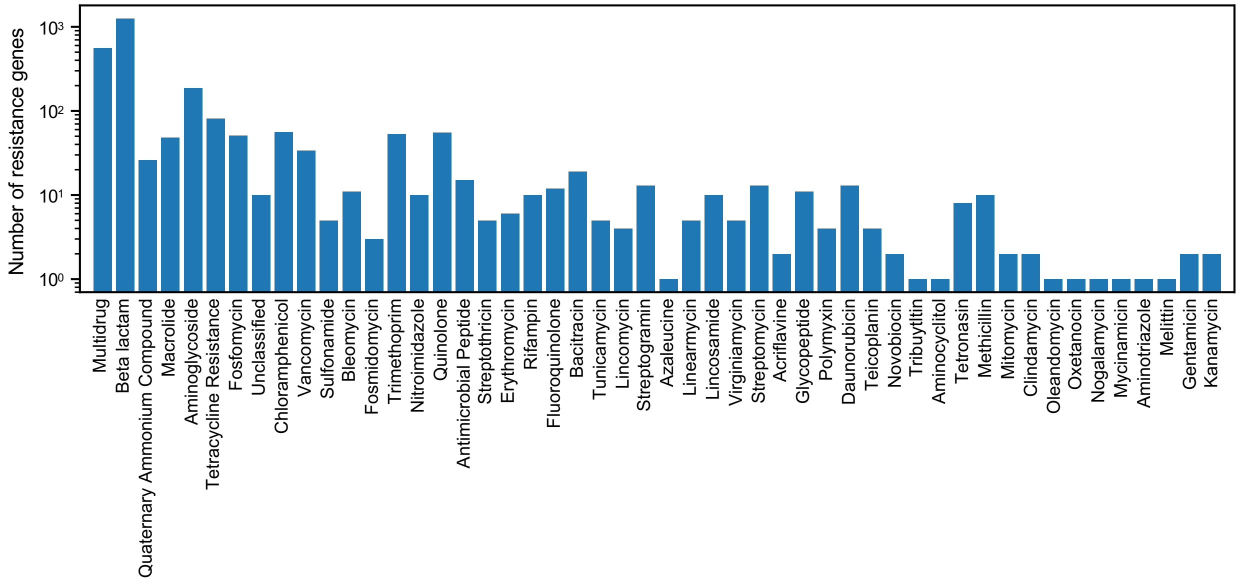

Appendix Figure S13. The number of different types of resistance genes in the pool of prokaryotic genes.

50 resistance types, including unclassified ARGs, were considered in the analysis.

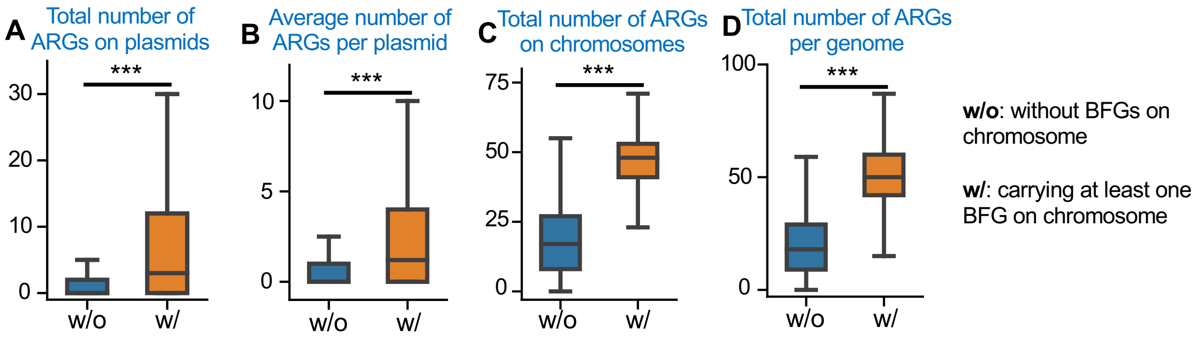

Appendix Figure S14. Carriage of BFGs on chromosomes is associated with enrichment of ARGs in prokaryotic genomes.

(A) The genomes with chromosomal BFGs are equipped with greater number of plasmid-borne ARGs. Here, only the genomes carrying at least one plasmid were considered in the comparison. The total number of plasmid-borne ARGs was calculated by summing all the ARGs on each plasmid in the genome. The triple asterisk notation (***) denotes statistical significance at the $p < 0.001$ level, as determined by two-sided Student's t-tests.

(B) Chromosomal BFG carriage brings about the enrichment of ARGs in each plasmid. Here, the average number of ARGs on each plasmid was calculated by normalizing the total number of ARGs by plasmid number in the genome. Only the genomes carrying at least one plasmid were considered in this analysis.

(C) The abundances of chromosomal ARGs are higher in genomes with chromosomal BFGs than in genomes without chromosomal BFGs.

(D) The total number of ARGs per genome, calculated by summing up ARGs in the chromosome and plasmids, is higher in genomes with chromosomal BFGs.

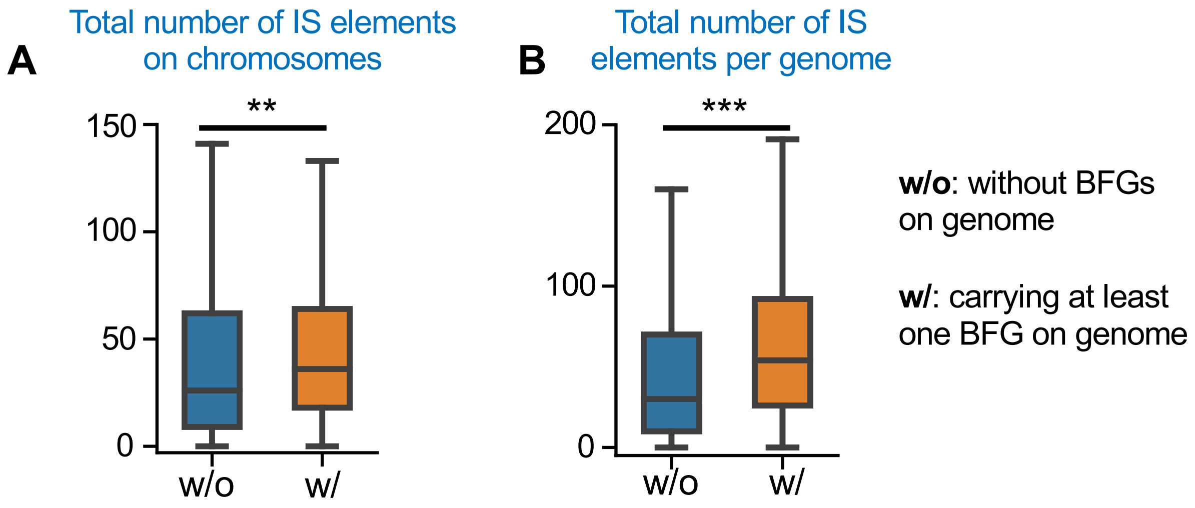

Appendix Figure S15. BFG carriage enriches the IS elements in prokaryotic genomes.

(A) The abundances of chromosomal IS elements in genomes with or without BFGs (p value = 0.0015). The double asterisk notation (**) denotes statistical significance at the $p < 0.01$ level, as determined by two-sided Student's t-tests.

(B) The total number of IS elements in genomes with or without BFGs. The triple asterisk notation (***) denotes statistical significance at the $p < 0.001$ level, as determined by two-sided Student's t-tests.

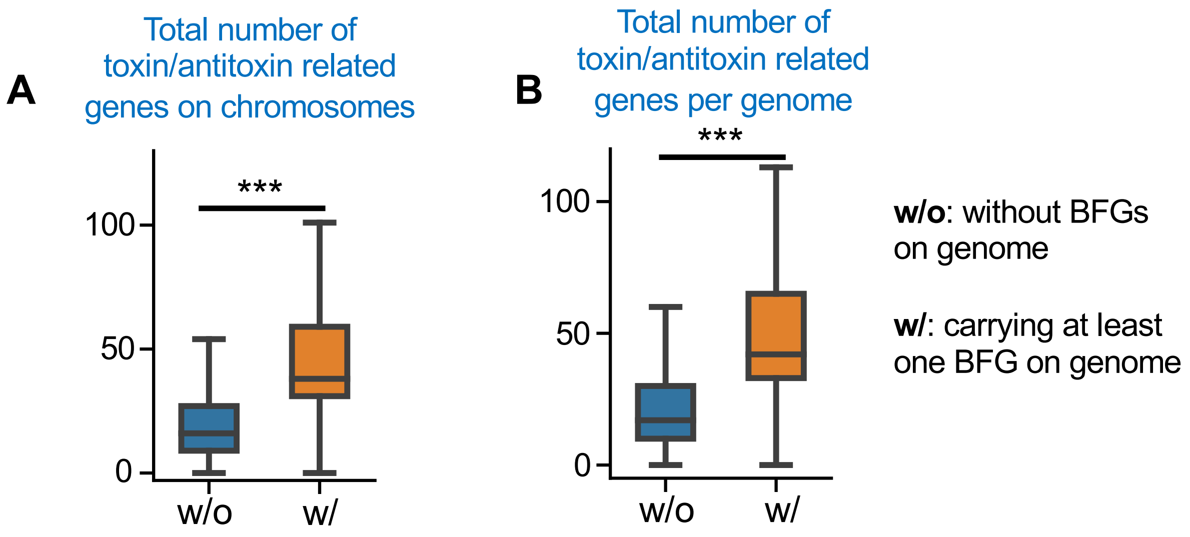

Appendix Figure S16. BFG carriage enriches toxin/antitoxin related genes in prokaryotic genomes.

(A) The abundances of chromosomal toxin/antitoxin related genes in genomes with or without BFGs. The triple asterisk notation (***) denotes statistical significance at the $p < 0.001$ level, as determined by two-sided Student's t-tests.

(B) The total number of toxin/antitoxin related genes in genomes with or without BFGs.

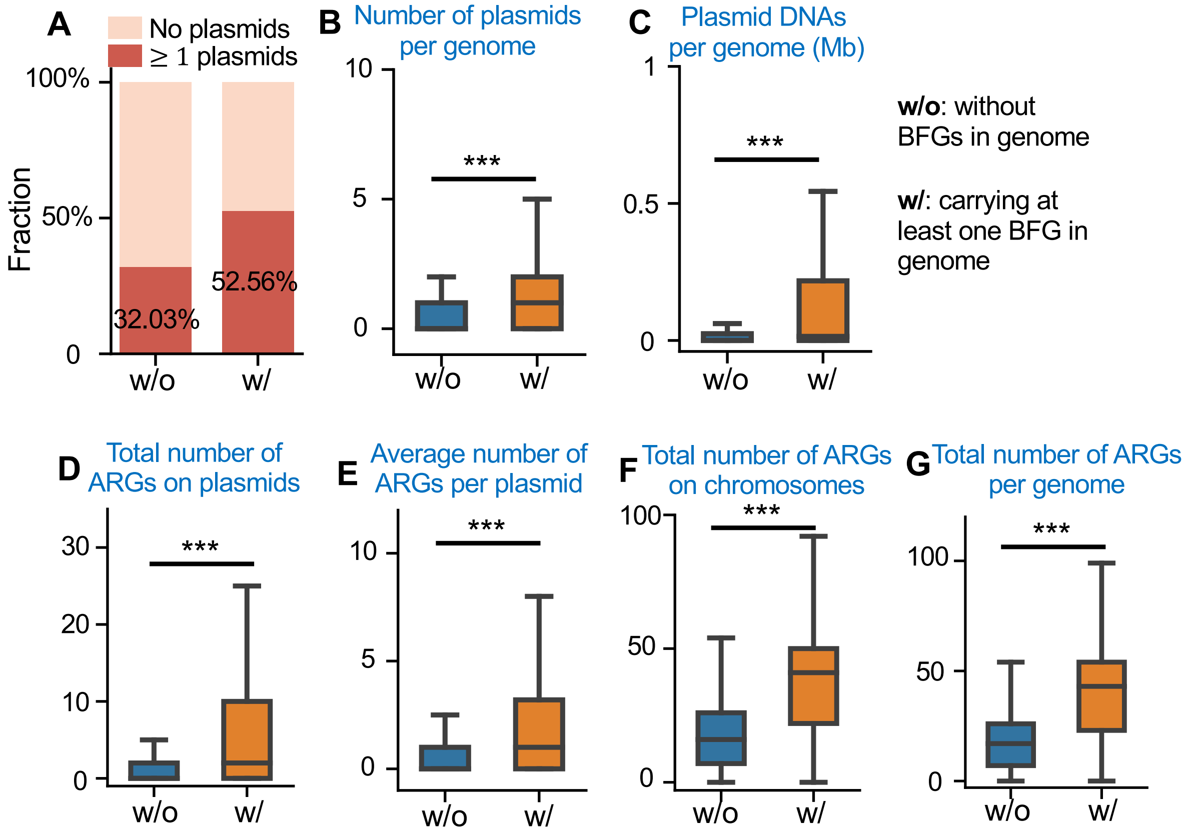

Appendix Figure S17. Biofilm formation remains positively correlated with the abundances of plasmids and ARGs in prokaryotic genomes, even with an expanded list of BFGs.

(A) Plasmid carriage in genomes with and without BFGs. 32.03% of genomes lacking biofilm-related genes carry plasmids, whereas this fraction increases to 52.56% in genomes with biofilm-related genes.

(B) Number of plasmids in genomes with and without BFGs. Genomes with BFGs generally carry a greater number of plasmids compared to those without BFGs. The triple asterisk notation (***) denotes statistical significance at the $p < 0.001$ level, as determined by two-sided Student's t-tests.

(C) Amount of plasmid DNA in genomes with and without BFGs. Genomes with BFGs carry a greater amount of plasmid DNA compared to those without BFGs. Here, the amount of plasmid DNA was calculated by summing the sizes of all different plasmids in the genome.

(D) Number of plasmid-borne ARGs in genomes with and without BFGs. Genomes with BFGs harbor a greater number of plasmid-borne ARGs. Only genomes carrying at least one plasmid were considered in this comparison. The total number of plasmid-borne ARGs was calculated by summing all ARGs on each plasmid in the genome.

(E) Enrichment of ARGs per plasmid in genomes with and without BFGs. BFG carriage leads to an enrichment of ARGs on each plasmid. The average number of ARGs per plasmid was calculated by normalizing the total number of ARGs by the number of plasmids in the genome. Only genomes carrying at least one plasmid were included in this analysis.

(F) Abundance of chromosomal ARGs in genomes with and without BFGs. The abundance of chromosomal ARGs is higher in genomes with BFGs compared to those without BFGs.

(G) Total number of ARGs per genome. The total number of ARGs per genome, calculated by summing ARGs in both chromosomes and plasmids, is higher in genomes with BFGs.

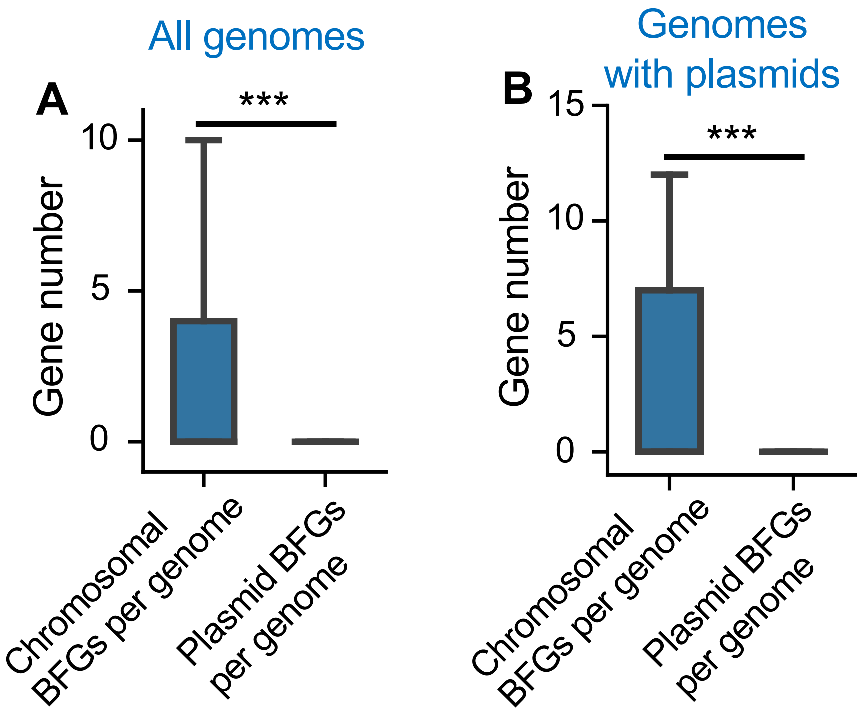

Appendix Figure S18. The majority of BFGs are carried by chromosomes instead of plasmids.

(A) The number of chromosomal or plasmid BFGs per genomes in all the prokaryotic genomes. The triple asterisk notation (***) denotes statistical significance at the $p < 0.001$ level, as determined by two-sided Student's t-tests.

(B) The number of chromosomal or plasmid BFGs per genomes in the prokaryotic genomes carrying at least one plasmid.

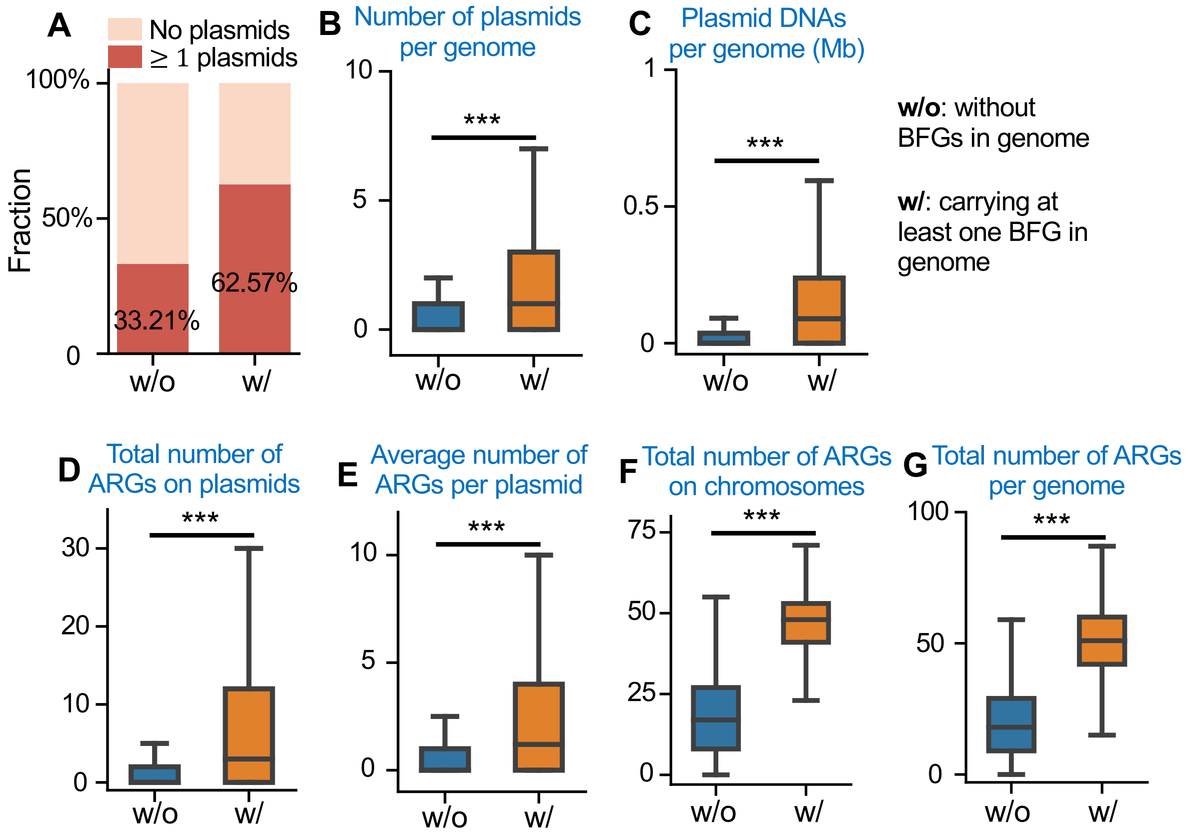

Appendix Figure S19. Biofilm formation remains positively correlated with the abundances of plasmids and ARGs in prokaryotic genomes, even when plasmids do not contribute to biofilm formation.

To assess the impact of plasmid-borne BFGs on our conclusions, genomes containing BFGs on plasmids were excluded from the analysis.

(A) Plasmid carriage in genomes with and without BFGs. 33.21% of genomes lacking biofilm-related genes carry plasmids, while this fraction increases to 62.57% in genomes with biofilm-related genes.

(B) Number of plasmids in genomes with BFGs. Genomes with BFGs generally carry a greater number of plasmids compared to those without BFGs. The triple asterisk notation (***) denotes statistical significance at the $p < 0.001$ level, as determined by two-sided Student's t-tests.

(C) Amount of plasmid DNA in genomes with BFGs. Genomes with BFGs carry a greater amount of plasmid DNA compared to those without BFGs. Here, the amount of plasmid DNA was calculated by summing the sizes of all different plasmids in the genome.

(D) Number of plasmid-borne ARGs in genomes with BFGs. Genomes with BFGs harbor a greater number of plasmid-borne ARGs. Only genomes carrying at least one plasmid were considered in this comparison. The total number of plasmid-borne ARGs was calculated by summing all ARGs on each plasmid in the genome.

(E) Enrichment of ARGs per plasmid in genomes with BFGs. BFG carriage leads to an enrichment of

ARGs on each plasmid. The average number of ARGs per plasmid was calculated by normalizing the total number of ARGs by the number of plasmids in the genome. Only genomes carrying at least one plasmid were included in this analysis.

(F) Abundance of chromosomal ARGs in genomes with BFGs. The abundance of chromosomal ARGs is higher in genomes with BFGs compared to those without BFGs.

(G) Total number of ARGs per genome. The total number of ARGs per genome, calculated by summing ARGs in both chromosomes and plasmids, is higher in genomes with BFGs.

Appendix Table S1. The ratio between cell mixture and LB media in different wells

		Wells										Spatial entropy
		1	2	3	4	5	6	7	8	9	10	
groups	A	1:9	1:9	1:9	1:9	1:9	1:9	1:9	1:9	1:9	1:9	1
	B	0:10	1:9	1:9	1:9	1:9	1:9	1:9	1:9	1:9	2:8	0.867
	C	0:10	0:10	1:9	1:9	1:9	1:9	1:9	1:9	1:9	3:7	0.712
	D	0:10	0:10	0:10	1:9	1:9	1:9	1:9	1:9	1:9	4:6	0.565
	E	0:10	0:10	0:10	0:10	1:9	1:9	1:9	1:9	1:9	5:5	0.438
	F	0:10	0:10	0:10	0:10	0:10	1:9	1:9	1:9	1:9	6:4	0.334
	G	0:10	0:10	0:10	0:10	0:10	0:10	1:9	1:9	1:9	7:3	0.251
	H	0:10	0:10	0:10	0:10	0:10	0:10	0:10	1:9	1:9	8:2	0.186

* The total volume in each well equaled 200 μL.

Appendix Table S2. List of biofilm formation-related genes curated from sequenced prokaryotic genomes

Index	Protein product	Gene
1	2'3' and 3'5' cyclic nucleotide monophosphates phosphodiesterase involved in biofilm formation	pdeB
2	ABC transporter (ATP-binding protein) biofilm formation	bifL
3	ABC transporter (permease) biofilm formation	bifM
4	acetate Na+-dependent symporter subunit involved in volatile signal for biofilm formation	vbfA
5	acetyl-glucosamine metabolite exporter component involved in biofilm formation	icaC
6	atypical membrane-integrating regulator of biofilm formation (Mistic protein)	mstX
7	bifunctional flagellar clutch and glycosyltransferase acting during biofilm formation	epsE
8	biofilm-associated Ig-like repeat protein Bap	bap
9	biofilm-associated Ig-like repeat protein Blp1	blp1
10	biofilm-associated metzincin protease inhibitor BamI	bamI
11	biofilm-associated protein	
12	biofilm-associated protein BapA	bapA
13	biofilm-dependent modulation protein	bdm
14	biofilm-forming protein	
15	biofilm-regulating peptide BriC	briC
16	biofilm-specific peroxidase AhpA	ahpA
17	biofilm-specific peroxidase; 2-cys peroxiredoxin	ahpA
18	biofilm-surface layer protein BslA	bslA
19	biofilm-surface layer protein BslB	bslB
20	biofilm architecture maintenance protein MbaA	mbaA
21	Biofilm associated protein A	
22	biofilm development protein AriR	
23	biofilm development protein YmgB/AriR	
24	biofilm development regulator YmgB/AriR family protein	
25	biofilm development YmgB/AriR family protein	
26	biofilm dispersion protein	bdlA
27	biofilm dispersion protein BdlA	bdlA
28	biofilm exopolysaccharide biosynthesis protein EpsG	epsG
29	biofilm extracellular matrix formation chain-length determining factor	epsG
30	biofilm formation methyltransferase WspC	wspC
31	biofilm formation protein MstX	mstX
32	biofilm formation protein PslA	pslA
33	biofilm formation protein PslB	pslB
34	biofilm formation protein PslC	pslC
35	biofilm formation protein PslD	pslD

36	biofilm formation protein PslE	pslE
37	biofilm formation protein PslF	pslF
38	biofilm formation protein PslG	pslG
39	biofilm formation protein PslH	pslH
40	biofilm formation protein PslI	pslI
41	biofilm formation protein PslJ	pslL
42	biofilm formation protein PslL	pslK
43	biofilm formation regulator BssR	bssR
44	biofilm formation regulator BssS	bssS
45	biofilm formation regulator diguanylate cyclase SiaD	siaD
46	biofilm formation regulator HmsP	hmsP
47	biofilm formation regulator kinase SiaB	siaB
48	biofilm formation regulator SiaD modulator protein SiaC	siaC
49	biofilm formation regulatory protein BssR	bssR
50	biofilm formation regulatory protein BssS	bssS
51	biofilm formation/cell division transcriptional regulator BrpA	brpA
52	biofilm forming exported protein	yoaW
53	biofilm hydrophobic layer component	bslA
54	biofilm master transcriptional regulator CsgD	csgD
55	biofilm matrix calcium-binding repeat protein CabA	cabA
56	biofilm matrix protein CalY	calY
57	biofilm matrix protein TasA	tasA
58	biofilm peroxide resistance protein	bsmA
59	biofilm peroxide resistance protein BsmA	bsmA
60	Biofilm PGA synthesis auxiliary protein PgaD	pgaD
61	biofilm PGA synthesis lipoprotein PgaB	pgaB
62	biofilm PGA synthesis N-glycosyltransferase PgaC	pgaC
63	biofilm PGA synthesis protein pgaA domain protein	
64	biofilm PGA synthesis protein PgaB	pgaB
65	biofilm PGA synthesis protein PgaC	pgaC
66	biofilm phosphatase Bph	bph
67	biofilm protein, member of the processed secretome contributing to biofilm hydrophobicity	bslB
68	biofilm regulation diguanylate cyclase SiaD	siaD
69	biofilm regulation phosphoprotein SiaC	siaC
70	biofilm regulation protein kinase SiaB	siaB
71	biofilm regulation protein phosphatase SiaA	siaA
72	biofilm regulator	bssS
73	biofilm regulator BssR	bssR
74	biofilm surface layer hydrophobin BslA	bslA

75	biofilm surface layer hydrophobin BslB	bslB
76	biofilm/acid-resistance regulator AriR	ariR
77	biofilm/acid-resistance regulator YmgB/AriR	
78	biofilm/motility transcriptional regulator AbfR1	abfR1
79	BsaA family SipW-dependent biofilm matrix protein	
80	c-di-GMP-binding biofilm dispersal mediator protein	bdcA
81	cell wall associated biofilm protein	
82	colanic acid and biofilm gene transcriptional regulator	mcbR
83	colanic acid/biofilm transcriptional regulator	
84	colanic acid/biofilm transcriptional regulator McbR	mcbR
85	DUF386 domain-containing toxin-antitoxin biofilm protein TabA	tabA
86	DVU1012 family biofilm structural adhesin	
87	DVU1545 family biofilm structural adhesin	
88	endocarditis and biofilm-associated pilus major subunit EbpC	ebpC
89	endocarditis and biofilm-associated pilus minor subunit EbpB	ebpB
90	endocarditis and biofilm-associated pilus tip protein EbpA	ebpA
91	essential sporulation DNA binding protein; regulator of biofilm formation	remA
92	extracellular matrix/biofilm regulator RemA	remA
93	LETM1-related biofilm-associated protein	
94	lipoprotein for biofilm formation	tapA
95	maintenance protein tyrosine kinase involved in biofilm formation	ptkA
96	major biofilm matrix component	tasA
97	master regulator for biofilm formation	
98	master regulator for biofilm formation via regulation of RNase Y (RicAFT complex / FAD / two [4Fe-4S]2+)	ricA
99	master regulator of biofilm formation	sinR
100	modulator of protein tyrosine kinase EpsB involved in biofilm matrix formation	epsA
101	modulator of PtkA protein tyrosine kinase activity; modulation of biofilm formation	tkmA
102	motility and biofilm regulator	glgS
103	pellicle/biofilm biosynthesis glycosyltransferase PelF	pelF
104	pellicle/biofilm biosynthesis outer membrane protein PelC	pelC
105	pellicle/biofilm biosynthesis protein PelB	pelB
106	pellicle/biofilm biosynthesis protein PelD	pelD
107	pellicle/biofilm biosynthesis protein PelE	pelE
108	pellicle/biofilm biosynthesis Wzx-like polysaccharide transporter PelG	pelG
109	potassium channel protein involved in biofilm formation	kbfO
110	protein tyrosine kinase involved in biofilm matrix formation	epsB
111	putative aminotransferase involved in biofilm matrix formation	epsN
112	putative biofilm synthesis domain protein	
113	putative biofilm synthesis protein	pru

114	putative exopolysaccharide pyruvyl transferase (biofilm formation)	yxaB
115	putative glycosyl transferase involved in biofilm matrix formation	epsJ
116	putative glycosyltransferase associated to biofilm formation	ydaM
117	putative glycosyltransferase involved in biofilm formation	epsH
118	putative methyl-tetrahydrofolate methyltransferase (biofilm formation)	yxjH
119	putative O-acetyltransferase involved in biofilm matrix formation	epsM
120	putative polysaccharide pyruvyl transferase involved in biofilm matrix formation	epsI
121	putative transcriptional regulator (GntR family, possibly involved in biofilm formation)	ymfC
122	putative UDP-sugar epimerase involved in biofilm matrix formation	epsC
123	pyruvyl transferase for matrix biofilm formation	epsO
124	RcsB connector protein for regulation of biofilm and acid-resistance	
125	regulator of biofilm formation	bssR
126	scyllo-inositol dehydrogenase (NADP+-dependent); biofilm formation	iolU
127	SipW-dependent biofilm matrix protein BsaA	bsaA
128	subunit of a sporulation, competence and biofilm formation regulatory complex controlling RNase Y (RicAFT complex / FAD / two [4Fe-4S]2+)	ricF
129	subunit of a sporulation, competence and biofilm formation regulatory complex of RNaseY (RicAFT complex / FAD / two [4Fe-4S]2+)	ricT
130	subunit of acetate transporter as a volatile signal for biofilm formation	vbfB
131	toxin-antitoxin biofilm protein TabA	tabA
132	transcriptional regulator of autolysin genes (biofilm formation)	slrR
133	transcriptional regulator of slrA (biofilm formation)	slrC
134	two-component response regulator [LinJ] (linearmycin resistance, biofilm formation)	linK
135	two-component sensor histidine kinase [LinK] (linearmycin resistance, biofilm formation)	linJ
136	two-component sensor potassium-responsive histidine kinase regulating cannibalism and biofilm formation	kinC
137	type IV pilus/biofilm regulator FimL	fimL

Appendix Table S3. List of biofilm formation-related genes curated from NCBI Gene database

Index	Protein product	Symbol
1	biofilm dispersion protein	bdlA
2	biofilm-dependent modulation protein	bdm
3	biofilm matrix protein TasA	tasA
4	biofilm formation protein PslD	pslD
5	pellicle/biofilm biosynthesis protein PelD	pelD
6	biofilm matrix calcium-binding repeat protein CabA	cabA
7	c-di-GMP-binding biofilm dispersal mediator protein	bdcA
8	biofilm formation/cell division transcriptional regulator BrpA	brpA
9	major biofilm matrix component	tasA
10	biofilm formation regulator BssS	bssS
11	biofilm formation stimulator Veg	veg
12	biofilm-associated protein BapA	bapA
13	regulator of biofilm formation	bssR
14	master regulator of biofilm formation	sinR
15	pellicle/biofilm biosynthesis Wzx-like polysaccharide transporter PelG	pelG
16	pellicle/biofilm biosynthesis outer membrane protein PelC	pelC
17	biofilm formation protein MstX	mstX
18	biofilm formation protein PslB	pslB
19	biofilm formation protein PslA	pslA
20	biofilm surface layer hydrophobin BslA	bslA
21	biofilm/acid-resistance regulator AriR	ariR
22	biofilm hydrophobic layer component	bslA
23	biofilm formation protein PslC	pslC
24	biofilm-associated Ig-like repeat protein Blp1	blp1
25	putative UDP-sugar epimerase involved in biofilm matrix formation	epsC
26	RbmA family biofilm matrix protein	F0316_RS09440
27	biofilm phosphatase Bph	bph
28	biofilm-regulating peptide BriC	briC
29	biofilm regulation protein phosphatase SiaA	siaA
30	pellicle/biofilm biosynthesis protein PelE	pelE
31	pellicle/biofilm biosynthesis glycosyltransferase PelF	pelF
32	extracellular matrix/biofilm regulator RemA	remA
33	biofilm-specific peroxidase AhpA	ahpA
34	biofilm master transcriptional regulator CsgD	csgD
35	endocarditis and biofilm-associated pilus major subunit EbpC	ebpC
36	endocarditis and biofilm-associated pilus minor subunit EbpB	ebpB

37	biofilm matrix protein CalY	calY
38	DUF386 domain-containing toxin-antitoxin biofilm protein TabA	tabA
39	2'3' and 3'5' cyclic nucleotide monophosphates phosphodiesterase involved in biofilm formation	pdeB
40	lipoprotein for biofilm formation	tapA
41	extracellular matrix/biofilm biosynthesis regulator RemA family protein	FLT43_RS16945
42	pellicle/biofilm biosynthesis protein PelB	pelB
43	type IV pilus/biofilm regulator FimL	fimL
44	endocarditis and biofilm-associated pilus tip protein EbpA	ebpA
45	biofilm surface layer hydrophobin BslB	bslB
46	biofilm formation regulator BssR	bssR
47	biofilm formation regulator HmsP	hmsP
48	biofilm exopolysaccharide biosynthesis protein EpsG	epsG
49	biofilm formation protein PslH	pslH
50	biofilm formation protein PslI	pslI
51	biofilm formation protein PslF	pslF
52	biofilm formation regulator kinase SiaB	siaB
53	biofilm formation regulator SiaD modulator protein SiaC	siaC
54	biofilm regulation diguanylate cyclase SiaD	siaD
55	biofilm regulation protein kinase SiaB	siaB
56	LETM1-related biofilm-associated protein	CA2559_RS12070
57	biofilm architecture maintenance protein MbaA	mbaA
58	biofilm development regulator YmgB/AriR family protein	SERAS9_RS14655
59	biofilm peroxide resistance protein BsmA	bsmA
60	toxin-antitoxin biofilm protein TabA	tabA
61	BsaA family SipW-dependent biofilm matrix protein	HDCHBGLK_RS13510
62	biofilm formation protein PslJ	pslJ
63	biofilm formation protein PslL	pslK
64	biofilm formation protein PslG	pslG
65	biofilm formation protein PslE	pslE
66	colanic acid/biofilm transcriptional regulator McbR	mcbR
67	maintenance protein tyrosine kinase involved in biofilm formation	ptkA
68	biofilm/acid-resistance regulator YmgB/AriR	A8F97_RS04455
69	biofilm dispersion protein BdlA	bdlA
70	biofilm regulation phosphoprotein SiaC	siaC

71	two-component sensor potassium-responsive histidine kinase regulating cannibalism and biofilm formation	kinC
72	biofilm formation methyltransferase WspC	wspC
73	atypical membrane-integrating regulator of biofilm formation (Mistic protein)	mstX
74	biofilm formation regulator diguanylate cyclase SiaD	siaD
75	transcriptional regulator of autolysin genes (biofilm formation)	slrR
76	bifunctional flagellar clutch and glycosyltransferase acting during biofilm formation	epsE
77	protein tyrosine kinase involved in biofilm matrix formation	epsB
78	acetate Na+-dependent symporter subunit involved in volatile signal for biofilm formation	vbfA
79	essential sporulation DNA binding protein; regulator of biofilm formation	remA
80	putative transcriptional regulator (GntR family, possibly involved in biofilm formation)	ymfC
81	biofilm forming exported protein	yoaW
82	putative glycosyltransferase associated to biofilm formation	ydaM
83	subunit of a sporulation, competence and biofilm formation regulatory complex controlling RNase Y (RicAFT complex / FAD / two [4Fe-4S]2+)	ricF
84	ABC transporter (permease) biofilm formation	bifN
85	master regulator for biofilm formation via regulation of RNase Y (RicAFT complex / FAD / two [4Fe-4S]2+)	ricA
86	two-component response regulator [LinJ] (linearmycin resistance, biofilm formation)	linK
87	potassium channel protein involved in biofilm formation	kbfO
88	biofilm-specific peroxidase; 2-cys peroxiredoxin	ahpA
89	putative glycosyltransferase involved in biofilm formation	epsH
90	modulator of protein tyrosine kinase EpsB involved in biofilm matrix formation	epsA
91	putative exopolysaccharide pyruvyl transferase (biofilm formation)	yxaB
92	putative methyltetrahydrofolate methyltransferase (biofilm formation)	yxjG
93	putative methyl-tetrahydrofolate methyltransferase (biofilm formation)	yxjH
94	transcriptional regulator of slrA (biofilm formation)	slrC
95	subunit of acetate transporter as a volatile signal for biofilm formation	vbfB
96	putative polysaccharide pyruvyl transferase involved in biofilm matrix formation	epsI
97	biofilm protein, member of the processed secretome contributing to biofilm hydrophobicity	bslB
98	scyllo-inositol dehydrogenase (NADP+-dependent); biofilm formation	iolU
99	putative glycosyl transferase involved in biofilm matrix formation	epsJ
100	biofilm extracellular matrix formation chain-length determining factor	epsG
101	subunit of a sporulation, competence and biofilm formation regulatory complex	ricT

	of RNaseY (RicAFT complex / FAD / two [4Fe-4S]2+)	
102	modulator of PtkA protein tyrosine kinase activity; modulation of biofilm formation	tkmA
103	putative O-acetyltransferase involved in biofilm matrix formation	epsM
104	putative aminotransferase involved in biofilm matrix formation	epsN
105	pyruvyl transferase for matrix biofilm formation	epsO
106	acetyl-glucosamine metabolite exporter component involved in biofilm formation	icaC
107	ABC transporter (ATP-binding protein) biofilm formation	bifL
108	two-component sensor histidine kinase [LinK] (linearmycin resistance, biofilm formation)	linJ
109	colanic acid and biofilm gene transcriptional regulator	mcbR
110	motility and biofilm regulator	glgS
111	biofilm peroxide resistance protein	bsmA
112	biofilm PGA synthesis N-glycosyltransferase PgaC	pgaC
113	biofilm regulator	bssS
114	SipW-dependent biofilm matrix protein BsaA	bsaA
115	Biofilm associated protein A	C1N69_RS21425
116	biofilm formation regulatory protein BssS	KPHS_19490
117	biofilm formation regulatory protein BssR	KPHS_17020
118	biofilm PGA synthesis lipoprotein PgaB	pgaB
119	putative biofilm synthesis protein	BDGL_001577
120	putative biofilm synthesis domain protein	BDGL_001573
121	colanic acid/biofilm transcriptional regulator	SF1765
122	Biofilm PGA synthesis auxiliary protein PgaD	BST28156_RS00710
123	biofilm-forming protein	SK061_RS13270
124	cell wall associated biofilm protein	CJZ72_RS03855

Appendix Table S4. List of adhesion-related genes curated from NCBI Gene database

Index	Protein product	Symbol
1	MCE family putative adhesion factor MAM7	mam7
2	migration/adhesion factor TmpA	tmpA
3	adhesion and penetration autotransporter App	app
4	adhesion and penetration autotransporter Hap	hap
5	adhesion-mediating acetaldehyde/alcohol dehydrogenase LAP	lap
6	ESA_00282 family adhesion-associated protein	JMV71_RS03730
7	adhesion protein FadA	RO08_RS09995
8	intracellular adhesion protein IcaD	icaD
9	adhesion domain-containing protein	SBG_RS11795
10	adhesion protein	PA2407
11	lipoprotein involved with copper homeostasis and adhesion	nlpE
12	adhesion component ABC transporter ATP-binding protein	Rv0986
13	adhesion component ABC transporter permease	Rv0987
14	intercellular adhesion protein C	SAOUHSC_03005
15	intercellular adhesion protein B	SAOUHSC_03004
16	copper homeostasis and adhesion lipoprotein	cutF
17	adhesion and penetration protein	SF1205
18	cell adhesion protein	QRY64_RS13225
19	invasin/intimin cell-adhesion domain protein	AACJ33_RS20710

Appendix Table S5. List of exopolysaccharide production-related genes curated from NCBI Gene database

Index	Protein product	Symbol
1	exopolysaccharide biosynthesis polyprenyl glycosylphosphotransferase	BPUM_RS15955
2	exopolysaccharide biosynthesis transcriptional regulator MucR	mucR
3	exopolysaccharide biosynthesis response regulator EpsW	epsW
4	PCP family exopolysaccharide biosynthesis protein EpsV	epsV
5	biofilm exopolysaccharide biosynthesis protein EpsG	epsG
6	exopolysaccharide biosynthesis transcriptional regulator SyrA	syrA
7	exopolysaccharide biosynthesis protein	ATU_RS06060
8	exopolysaccharide biosynthesis glycosyltransferase VpsL	vpsL
9	exopolysaccharide biosynthesis beta-barrel protein VpsM	vpsM
10	exopolysaccharide biosynthesis glycosyltransferase EpsA	epsA
11	exopolysaccharide biosynthesis glycosyltransferase EpsD	epsD
12	exopolysaccharide biosynthesis GT4 family glycosyltransferase EpsE	epsE
13	exopolysaccharide biosynthesis GT2 family glycosyltransferase EpsU	epsU
14	exopolysaccharide biosynthesis flippase	wzx
15	exopolysaccharide biosynthesis polyisoprenyl-phosphate hexose-1-phosphate transferase EpsZ	epsZ
16	exopolysaccharide biosynthesis protein VpsQ	vpsQ
17	exopolysaccharide biosynthesis protein VpsP	vpsP
18	exopolysaccharide biosynthesis protein VpsJ	vpsj
19	exopolysaccharide biosynthesis protein VpsH	vpsH
20	exopolysaccharide biosynthesis glycosyltransferase VpsK	vpsK
21	exopolysaccharide biosynthesis glycosyltransferase EpsH	epsH
22	exopolysaccharide biosynthesis acetyltransferase VpsC	vpsC
23	exopolysaccharide biosynthesis flippase VpsE	vpsE
24	exopolysaccharide biosynthesis protein VpsF	vpsF
25	exopolysaccharide biosynthesis acetyltransferase VpsG	vpsG
26	exopolysaccharide biosynthesis glycosyltransferase VpsI	vpsI
27	exopolysaccharide biosynthesis glycosyltransferase VpsD	vpsD
28	exopolysaccharide production protein ExoY	exoY
29	exopolysaccharide production repressor ExoX	exoX
30	exopolysaccharide production protein ExoZ	exoZ
31	exopolysaccharide production regulator ExoR	exoR
32	exopolysaccharide production protein ExoF	exoF
33	exopolysaccharide production protein YjbE	yjbE
34	exopolysaccharide production repressor protein	ATU_RS18975
35	exopolysaccharide production repressor ExoX protein	A4R29_RS02260
36	exopolysaccharide production protein	FB462_RS12005

Appendix Table S6. List of extracellular matrix-related genes curated from NCBI Gene database

Index	Protein product	Symbol
1	extracellular matrix protein-binding adhesin Emp	emp
2	extracellular matrix/biofilm regulator RemA	remA
3	extracellular matrix/biofilm biosynthesis regulator RemA family protein	FLT43_RS16945
4	extracellular matrix regulator RemB	remB
5	extracellular matrix and plasma binding protein	SAOUHSC_00816
6	putative extracellular matrix component exporter; putative cyclic di-GMP receptor	epsK
7	regulator of extracellular matrix formation	remB
8	putative extracellular matrix glycosyltransferase	epsD
9	biofilm extracellular matrix formation chain-length determining factor	epsG
10	putative phosphotransferase involved in extracellular matrix synthesis	epsL

Appendix Table S7. List of quorum sensing-related genes curated from NCBI Gene database

Index	Protein product	Symbol
1	quorum-sensing control repressor	qscR
2	quorum sensing protein	luxS
3	HTH-type quorum sensing-dependent transcriptional regulator VjbR	vjbR
4	quorum sensing response regulator transcription factor QseB	qseB
5	quorum sensing histidine kinase QseC	qseC
6	quorum-sensing system transcriptional regulator Rgg3	rgg3
7	quorum-sensing system transcriptional regulator Rgg2	rgg2
8	quorum-sensing master transcriptional regulator HapR	hapR
9	quorum-sensing sigma-54 dependent transcriptional regulator LuxO	luxO
10	LuxR/HapR/OpaR family quorum-sensing transcriptional regulator	BCT95_RS01900
11	quorum-sensing phosphorelay protein LuxU	luxU
12	quorum-sensing response regulator AgrA	EQ029_RS04440
13	quorum-sensing peptide PapR	papR
14	alpha-hydroxyketone-type quorum-sensing autoinducer synthase	cqsA
15	quorum-sensing system transcriptional regulator Rgg	rgg
16	quorum-sensing transcriptional repressor QscR	qscR
17	quorum-sensing sensor histidine kinase AgrC	MUA40_RS03975
18	PhrA family quorum-sensing system peptide	SPPN_RS14240
19	quorum-sensing CAI-1 autoinducer sensor kinase/phosphatase CqsS	cqsS
20	quorum-sensing transcriptional regulator RsaL	rsaL
21	HTH-type quorum sensing-dependent transcriptional regulator RpaR	rpaR
22	two-component response quorum-sensing regulator	comA
23	putative quorum-sensing-regulated virulence factor	PNUC_RS03650
24	PA3611 family quorum-sensing-regulated virulence factor	HLB40_RS06140
25	quorum-sensing autoinducer 2 sensor kinase/phosphatase LuxQ	luxQ
26	quorum-sensing autoinducer LAI-1 synthase LqsA	lqsA
27	quorum-sensing system pheromone BlpC	blpC
28	quorum-sensing-regulated virulence factor family protein	ELZ14_RS06150
29	quorum-sensing autoinducer synthase	F460_RS0116645
30	secreted inhibitor of the activity of phosphatase RapA (quorum sensing)	phrA
31	quorum sensing sensory histidine kinase in two-component regulatory system with QseB	qseC
32	quorum sensing DNA-binding response regulator in two-component regulatory system with QseC	qseB
33	quorum-sensing transcriptional activator	sdiA
34	quorum-sensing system DWW-type pheromone	EL097_RS10825
35	quorum-sensing system protein StcA	stcA
36	uncharacterized LOC11432379	LOC11432379

37	transcriptional activator of quorum sensing autoinducer synthesis transcription regulator protein	solR
38	putative quorum sensing peptide	P9653_gp41
39	AimR-like quorum sensing transcription regulator	P9653_gp40
